# Modulation of cell differentiation and growth underlies the shift from bud protection to light capture in cauline leaves

**DOI:** 10.1093/plphys/kiae408

**Published:** 2024-08-06

**Authors:** Constance Le Gloanec, Andrea Gómez-Felipe, Viraj Alimchandani, Elvis Branchini, Amélie Bauer, Anne-Lise Routier-Kierzkowska, Daniel Kierzkowski

**Affiliations:** Département de Sciences Biologiques, Institut de Recherche en Biologie Végétale, Université de Montréal, 4101 Sherbrooke St E, Montréal, QC H1X 2B2, Canada; Département de Sciences Biologiques, Institut de Recherche en Biologie Végétale, Université de Montréal, 4101 Sherbrooke St E, Montréal, QC H1X 2B2, Canada; Département de Sciences Biologiques, Institut de Recherche en Biologie Végétale, Université de Montréal, 4101 Sherbrooke St E, Montréal, QC H1X 2B2, Canada; Département de Sciences Biologiques, Institut de Recherche en Biologie Végétale, Université de Montréal, 4101 Sherbrooke St E, Montréal, QC H1X 2B2, Canada; Département de Sciences Biologiques, Institut de Recherche en Biologie Végétale, Université de Montréal, 4101 Sherbrooke St E, Montréal, QC H1X 2B2, Canada; Département de Sciences Biologiques, Institut de Recherche en Biologie Végétale, Université de Montréal, 4101 Sherbrooke St E, Montréal, QC H1X 2B2, Canada; Département de Sciences Biologiques, Institut de Recherche en Biologie Végétale, Université de Montréal, 4101 Sherbrooke St E, Montréal, QC H1X 2B2, Canada

## Abstract

Plant organs have evolved into diverse shapes for specialized functions despite emerging as simple protrusions at the shoot apex. Cauline leaves serve as photosynthetic organs and protective structures for emerging floral buds. However, the growth patterns underlying this dual function remain unknown. Here, we investigate the developmental dynamics shaping Arabidopsis (*Arabidopsis thaliana*) cauline leaves underlying their functional diversification from other laminar organs. We show that cauline leaves display a significant delay in overall elongation compared with rosette leaves. Using live imaging, we reveal that their functional divergence hinges on early modulation of the timing of cell differentiation and cellular growth rates. In contrast to rosette leaves and sepals, cell differentiation is delayed in cauline leaves, fostering extended proliferation, prolonged morphogenetic activity, and growth redistribution within the organ. Notably, cauline leaf growth is transiently suppressed during the early stages, keeping the leaf small and unfolded during the initiation of the first flowers. Our findings highlight the unique developmental timing of cauline leaves, underlying their shift from an early protective role to a later photosynthetic function.

## Teaser

Cell differentiation delay, growth redistribution, and transient growth decrease during development ensure the dual function of the cauline leaf in protection and light capture.

## Introduction

Plant lateral organs, such as leaves and flowers, exhibit an incredible diversity of shapes that evolved to ensure their specialized functions and to adapt to their environment. For instance, leaves of different species range from simple to compound with marginal protrusions of varying sizes and shapes (Fukushima and Hasebe 2014; Vlad et al. 2014). Leaf diversity may also be observed within individual plants as leaf morphology changes during overall plant development—a process called heteroblasty (Nikovics et al. 2006; Yu et al. 2015; Maugarny-Calès and Laufs 2018; Tang et al. 2023; Li et al. 2024). Furthermore, other laminar organs, such as sepals and petals, differ greatly from leaves in their forms and sizes despite deriving from a leaf-like ancestral structure (Bowman et al. 1991; Honma and Goto 2001; Pelaz et al. 2001).

Regardless of their diversity at maturity, all aerial organs are initiated as simple protrusions at the shoot apical meristem (Hervieux et al. 2016; Echevin et al. 2019; Burian et al. 2022; Silveira et al. 2022). All of them are also suggested to follow a common developmental program after initiation (Burko and Ori 2013; Runions et al. 2017; Whitewoods et al. 2020; Challa et al. 2021). For instance, studies on leaf diversification revealed common growth behaviors at the global and local scales in both simple and compound leaves (Ori et al. 2007; Kierzkowski et al. 2019). Early quantitative alterations of this shared developmental mechanism are believed to account for the strong differences in final leaf shapes (Kierzkowski et al. 2019; Whitewoods et al. 2020; Wang et al. 2022). How this common program is fine-tuned to achieve specific shapes at maturity is still unclear, but involves precise molecular tuning of patterning, growth, and differentiation (Sablowski 2015; Kierzkowski et al. 2019; Hamant and Saunders 2020; Whitewoods et al. 2020; Tang et al. 2023).

Current evidence suggests that the balance between proliferative growth and differentiation is essential in determining the geometry of laminar organs. For instance, the rate of margin differentiation plays a significant role in shaping leaf forms, with delayed differentiation enabling extended patterning and greater leaf complexity (Donnelly et al. 1999; Shani et al. 2010; Bar and Ori 2014; Vuolo et al. 2018; Kierzkowski et al. 2019). Local auxin maxima at the margin, facilitated by the PIN-FORMED1 (PIN1) auxin transporter, lead to the formation of protrusions such as serrations or leaflets. However, only undifferentiated cells at the leaf margin are competent to generate auxin maxima and respond to auxin by locally increasing and reorienting growth (Ori et al. 2007; Barkoulas et al. 2008; Bilsborough et al. 2011; Kasprzewska et al. 2015; Ben-Gera et al. 2016; Kierzkowski et al. 2019).

Auxin distribution at the margin has also been suggested to act globally by coordinating cellular growth orientations (i.e. growth anisotropy). In leaves, a distal auxin maximum correlates with global growth orientations converging toward the leaf tip, likely underlying organ shape tapering toward the tip (Jaeger et al. 2008; Kuchen et al. 2012). Conversely, petals exhibit a broader pattern of auxin distribution at the margin, which likely leads to the diverging growth anisotropy underlying its distally broadening shape (Green et al. 2010; Lampugnani et al. 2013; Sauret-Güeto et al. 2013). Thus, auxin plays a critical role in guiding organ formation, although the extent to which it operates locally or globally is still a matter of debate (Bilsborough et al. 2011; Kuchen et al. 2012; Kierzkowski et al. 2019; Whitewoods et al. 2020).

Understanding how plant organs acquire their shapes is essential in the context of their specific functions. For instance, in Arabidopsis (*Arabidopsis thaliana*), rosette leaves are large organs optimized for photosynthesis, while sepals are small, protecting the internal floral organs during their development (Roeder et al. 2010; Rodriguez et al. 2014; Bielczynski et al. 2017). Cauline leaves are the last few leaves initiated during the meristem transition from the vegetative to the reproductive phase. On one hand, they are efficient photosynthetic organs, contributing to the plant's overall energy production (Su et al. 2011). However, they also serve as protective structures for emerging floral buds during early bolting stages (Ding et al. 2023; Pabõn-Mora et al. 2013). This suggests that the development of these leaves might exhibit characteristics of both rosette leaves and laminar organs of the flower such as sepals (Hempel and Feldman 1994; Yang and Jiao 2016). Indeed, when floral identity genes are ectopically expressed in *Arabidopsis*, cauline leaves convert into petal-like organs, while rosette leaf development is unaffected (Krizek and Meyerowitz 1996; Pelaz et al. 2001). Despite these intriguing features, our understanding of the developmental dynamics of cauline leaves remains unknown.

Here, we characterize the developmental trajectories contributing to the formation of cauline leaves underlying their functional diversification from rosette leaves and sepals in the model species *A. thaliana*. We show that cauline leaves display a strong delay in overall elongation compared with rosette leaves. Through quantitative live imaging, we demonstrate that cauline leaf functional divergence mainly relies on the early modulation of two key components: (i) the rate and distribution of cellular growth, and (ii) the timing of cell differentiation. In contrast to rosette leaves and sepals, cell differentiation is strongly delayed in the cauline leaf, allowing extended cell proliferation in the leaf blade, prolonged morphogenetic activity at the margin, and growth redistribution within the developing organ. Importantly, cauline leaf growth is transiently suppressed at very early developmental stages, allowing the leaf to stay small and unfolded during the initiation of the first flowers. Overall, our results demonstrate the unique developmental trajectory of the cauline leaf that underlies the transition from its early protective role to the late photosynthetic function.

## Results

### Elongation and unfolding of the cauline leaves are delayed compared with rosette leaves

**Figure 1.**
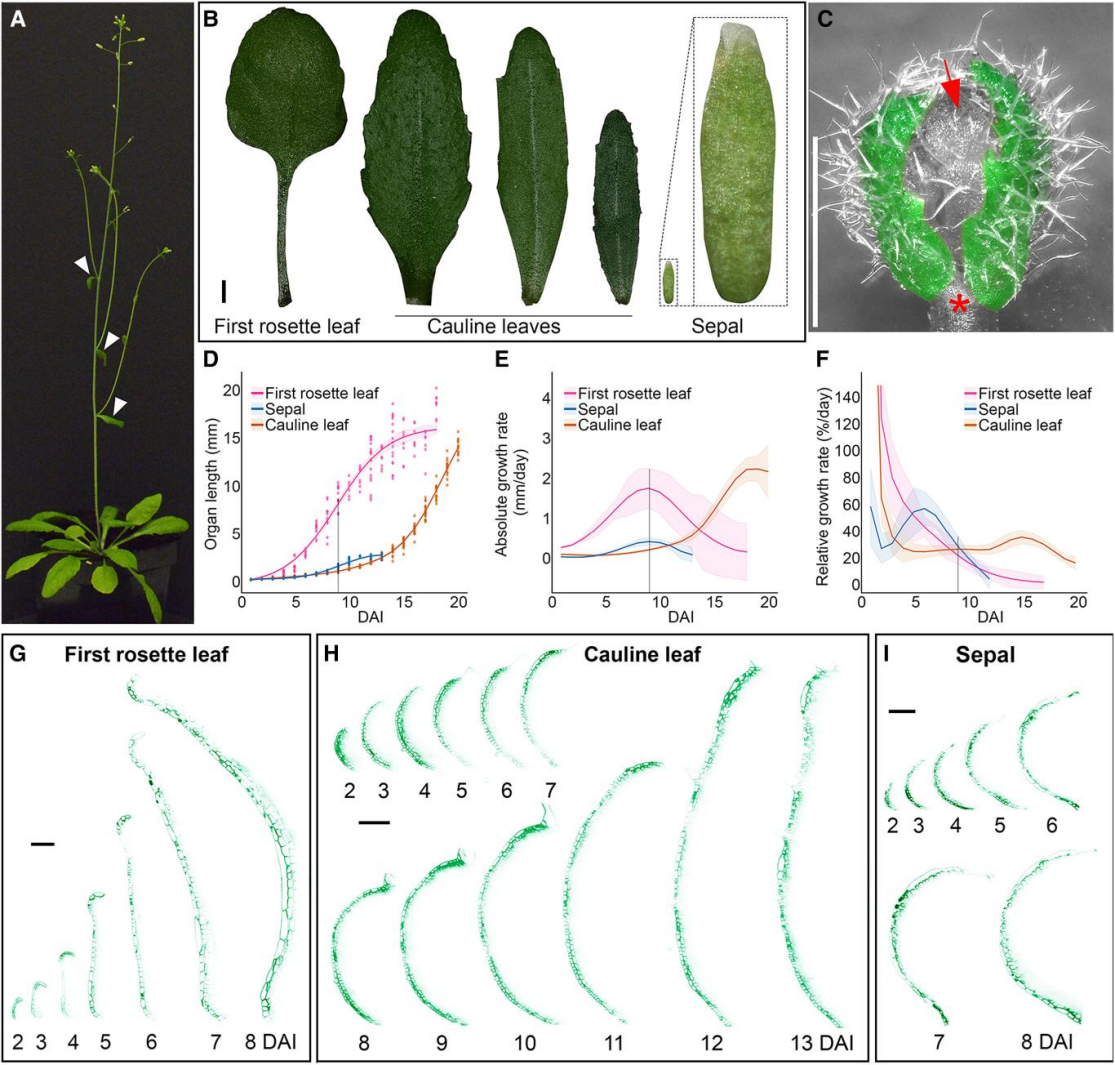
Elongation and unfolding of cauline leaves are delayed compared with rosette leaves. **A)** Four-week-old *Arabidopsis thaliana* plant. Cauline leaves are indicated by white arrowheads. **B)** First rosette leaf (left), cauline leaves (middle), and sepal (right) at maturity. Inset: close-up view of the sepal. Images were digitally extracted for comparison. **C)** Cauline leaves (colored in green) cover the inflorescence meristem and developing floral buds (red arrow). Star indicates the removed cauline leaf to uncover the initiating flowers. Note the high density of trichomes on the abaxial surface. **D)** Organ length plotted against time from initiation to maturity. Points represent independent measurement (*n* = 4 to 21). **E)** Organ absolute elongation rate. **F)** Organ relative elongation rate, showing a clear resurgence of growth in the cauline leaf at later stages. **D to F)** The gray line indicates the time of peak absolute growth rate for the first rosette leaf and the sepal, at 9 DAI. **G to I)** Digital, longitudinal sections located in the medial part of the developing first rosette leaf **G)**, cauline leaf **H)**, and sepal **I)**. Abaxial sides of each organ (shown in green) is oriented toward the left side of each series. DAI indicates days after primordium initiation. Scale bars: 1 mm in **B** and **C** and 100 *µ*m in **G** to **I**. See also Supplementary Fig. S1.

Cauline leaves exhibit both vegetative and floral features, as they are initiated from the reproductive meristem (Hempel and Feldman 1994; Pastore et al. 2011). In contrast to rosette leaves, which have petioles for optimizing light capture, cauline leaves lack petioles but efficiently perform photosynthesis as they are located on the stem, so have direct access to light without being in the shade of other leaves (Fig. 1A and B) (Ding et al. 2023). While at early developmental stages, all leaves protect fragile meristems, cauline leaves have been suggested to serve this function in an analogous way to sepals, which shield developing internal organs of flowers for an extended period (Fig. 1C) (Pabõn-Mora et al. 2013; Ding et al. 2023). The presence of abaxial trichomes on cauline leaves reinforces their protective function (Fig. 1C) (Karabourniotis et al. 2020; Berhin et al. 2021).

We supposed that the dual role of cauline leaves could be reflected in their growth dynamics. Therefore, we characterized the overall elongation of cauline leaves throughout their development and compared them with first rosette leaves and sepals. We derived two different measures of organ growth. The *absolute* elongation rate (in millimeter per day) simply reflects the increment of organ length over time (Fig. 1E). The *relative* elongation rate (in percent per day) shows how fast the organ elongates in proportion to its previous length (Fig. 1F). While the absolute elongation rate is more intuitive than the relative rate, the second measures growth independently from organ size and reflects better cell wall expansion.

The length of first rosette leaves evolved over time following a characteristic sigmoid shape (Fig. 1D), consistent with previous studies on leaf elongation (Cookson and Granier 2006; Massonnet et al. 2010; Baerenfaller et al. 2015). The speed at which the first leaf elongated peaked at 9 d after initiation [absolute growth rate (AGR); 1.8 mm per day ± 0.5 SE at 9 DAI] (Fig. 1E). This peak corresponded to the middle of the sigmoid curve when the first leaf reached about half its final size and experienced a linear growth phase (Fig. 1D). Splitting our leaf measurements into petiole and blade length (Supplementary Fig. S1) showed that they followed slightly different sigmoid functions, with the peak of absolute elongation occurring faster in the blade (at 8 DAI) than in the petiole (at 11 DAI), which grew slower but for a longer time (Supplementary Fig. S1A). In contrast with the measurements of absolute elongation, the relative elongation rate was maximal on the very first days after leaf initiation and decreased rapidly afterward, reaching around 20% per day at 9DAI (Fig. 1F and Supplementary Fig. S1C). This fast decrease confirmed previous studies on rosette leaves (Kierzkowski et al. 2019; Le Gloanec et al. 2022; Harline and Roeder 2023).

Growth dynamics in sepals showed similarities with the first rosette leaves, with maximal values of relative elongation rate in the days just after initiation, followed by a fast decrease (Fig. 1F). Sepals experienced their peak of absolute elongation at 9 DAI, like the first leaves, although with a much lower rate (0.42 mm per day ± 0.09 SE) (Fig. 1E), which explains the smaller length of sepals at maturity (2.6 mm ± 0.2 SE). Ultimately, the low peak of absolute elongation in sepals is due to the slower relative elongation before 9 DAI, preventing the sepals from accumulating length early on (Fig. 1D).

Curiously, cauline leaves displayed a very different growth trajectory. Cauline leaves maintained a smaller size than sepals until 13 DAI (Fig. 1D), due to a very low absolute elongation rate (Fig. 1E). This was caused by a sharp decrease in relative elongation just after initiation and maintenance of a low relative growth rate (RGR) (around 25% per day) between 4 and 12 DAI (Fig. 1F). The first leaves and sepals reached their final size around 13 DAI, at which point they ceased growing both in absolute and relative terms. By contrast, around 13 DAI, the cauline leaf increased its growth rates, reaching a peak in relative elongation rate at 15 DAI (35% per day ± 5 SE), followed by a peak in absolute elongation at 19 DAI. This late growth acceleration compensated for early slow growth, allowing cauline leaves to reach the size of the first rosette leaves by 21 DAI (Fig. 1D).

This transition in growth dynamics may reflect the dual function of the cauline leaf, starting with an early protective role followed by the acquisition of photosynthetic competency. If this is true, the geometry of the cauline leaves at early developmental stages should resemble that observed in sepal. To test this hypothesis, we monitored the early primordia development using time-lapse imaging starting from two days after initiation, when all of them have comparable shape and size (refer to the “Materials and methods” section for details) (Fig. 1, G to I). While the sepal remained curved over the floral bud (Fig. 1I), the first rosette leaf began unfolding around 4 DAI (Fig. 1G), at the same time as its absolute elongation rate started to increase (Fig. 1E). The cauline leaf remained curled toward the floral bud until 12 to 13 DAI (Fig. 1H), which also coincides with the onset of its faster absolute elongation (Fig. 1E). At their early stages of development, both the first rosette leaf and the cauline leaf could protect their respective meristems. Both organs change their curvature and potentially capture light once entering their fast phase of absolute elongation. The delayed peak of the absolute elongation rate for the cauline leaf could help maintain its protective role for an extended period.

### Cauline leaves display two successive waves of growth at the cellular scale

We have shown that, similarly to the sepals, in the few days after initiation cauline leaves display slower relative elongation rates than rosette leaves. Subsequently, the relative elongation of cauline leaves accelerates (Fig. 1F), allowing them to catch up in size with rosette leaves (Fig. 1D). To further understand this divergence at the cellular scale, we used our confocal time-lapse imaging pipeline to compute cell area expansion and cell elongation (in relative terms) along the proximodistal and mediolateral organ axes for each cell in the abaxial epidermis using the MorphoGraphX software (Strauss et al. 2022).

**Figure 2.**
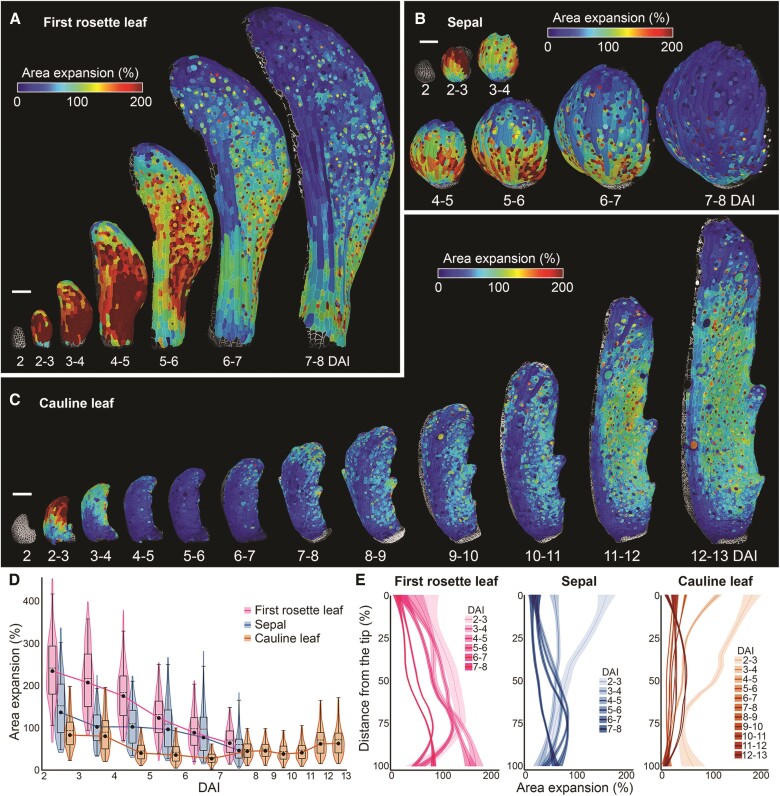
Cauline leaf displays two successive waves of growth. **A to C)** Heat maps of area expansion for the *Arabidopsis thaliana* first rosette leaf **A)**, sepal **B)**, and cauline leaf **C)**. Heat maps generated between two consecutive time points are displayed on the digitally extracted organ surface at the later time point. **D)** Quantifications of area expansion for observed laminar organs. Violin plots and boxplots represent 90% of the values; dashed lines represent mean, black dots median, boxes interquartile range, and whiskers 90% of data (*n* = 810 to 5,827 cells, three independent time-lapse series). **E)** Quantifications of area expansion along the proximodistal axis of the first rosette leaf (left), sepal (middle), and cauline leaf (right). Ribbon plots display the normalized distance; the mean is represented by a line, standard deviation by the shaded area (*n* = 58 to 2,626 cells, based on time-lapse series shown in **A** to **C**). DAI indicates days after primordium initiation. Scale bars: 100 *µ*m. See also Supplementary Fig. S2.

Just after initiation (2 to 3 DAI), relative cellular growth in all organs was fast, with the highest growth rates registered in the abaxial epidermis of the first rosette leaf (Fig. 2A to D; Supplementary Fig. S2). In the cauline leaf and sepal, cells located at the organ tip grew the fastest, while at this stage, cellular growth rates were more homogeneous in the first rosette leaf (Fig. 2). From 3 to 7 DAI, we have observed a progressive decrease in growth rates in all organs (Fig. 2A to E). During this phase, a clear basipetal (from tip to base) gradient of growth was established both in the first rosette leaf and sepal (Fig. 2A, B, D, and E; Supplementary Fig. S2A, C, D, and F), while in the cauline leaf, cellular growth became very low and homogenous all over the abaxial epidermis (Fig. 2C to E; Supplementary Fig. S2B and E). After 6 DAI, the growth rate further decreased in the first rosette leaf and sepal (Fig. 2A, B, D, and E). By contrast, after a period of minimal growth, the cauline leaf slightly accelerated its cellular expansion from around 8 DAI (Fig. 2C and D). Interestingly, cells located at the tip of this leaf were the first to increase their growth rate, especially along the longitudinal axis of the organ (Fig. 2C to E; Supplementary Fig. S2B and E). A typical basipetal growth gradient started to be visible in the cauline leaf only from around 11 DAI when cells at the leaf tip stopped expanding (Fig. 2C to E; Supplementary Fig. S2B and E). Overall, these results show that the growth rates of abaxial epidermal cells at early developmental stages of the cauline leaf are much slower than in the rosette leaves. As a result of this early decrease in growth, the cauline leaf transiently maintains a size in the range observed for sepals. Remarkably, while rosette leaves and sepals progressively slow down growth over their development, the cauline leaf increases its growth at later stages. Thus, two successive waves of growth in the cauline leaf seem to enable its dual function.

### Cauline leaf epidermis maintains cell divisions during the early and late growth phases

**Figure 3.**
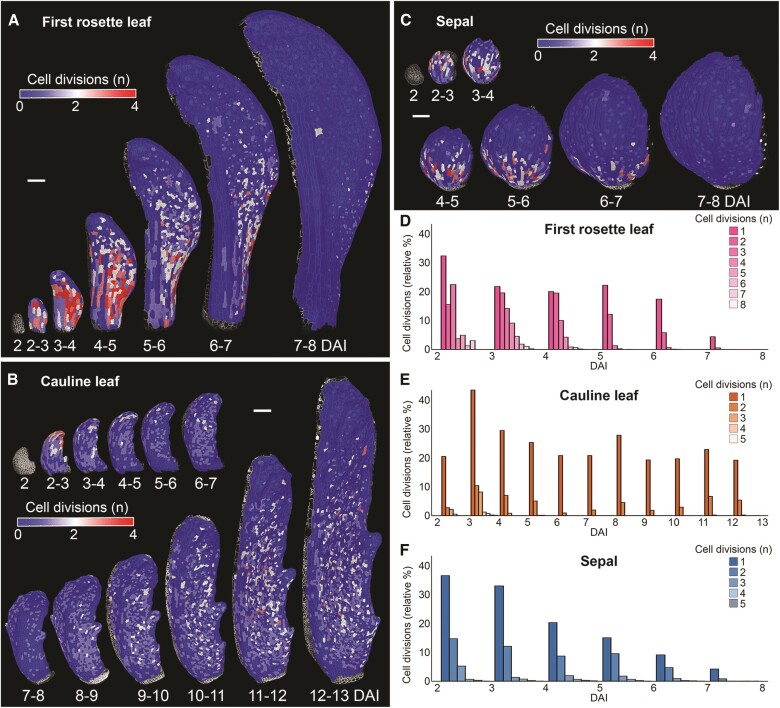
Cauline leaf maintains cell divisions during early and late growth phases. **A to C)** Heat maps of cell divisions for the *Arabidopsis thaliana* first rosette leaf **A)**, cauline leaf **B)**, and sepal **C)**. Heat maps generated between two consecutive time points are displayed on the digitally extracted organ surface at the later time point. **D to F)** Quantifications of cell divisions in first rosette leaf **D)**, cauline leaf **E)**, and sepal **F)**. Bar plots represent the relative proportion of cell divisions, normalized by the total number of cells (*n* = 812 to 5,828 cells, three independent time-lapse series). DAI indicates days after primordium initiation. Scale bars: 100 *µ*m.

During aerial organ morphogenesis, cells located in fast-growing tissues usually divide more frequently compared with slow-growing areas (Andriankaja et al. 2012; Fox et al. 2018; Zhang et al. 2020; Le Gloanec et al. 2022; Harline and Roeder 2023). The basipetal growth decrease in both the first rosette leaf and sepal strongly correlated with the decrease of cell divisions from the organ tip to the base (Figs. 2A, B and 3A, C). In both organs, cell proliferation activity was first very intensive but quickly decreased at later stages (8 DAI), consistent with a progressive slowdown of growth (Figs. 2A, B, D and 3A, C, D, and F). By contrast, the cauline leaf epidermis retained its cell proliferative activity until at least 13 DAI, correlating with its prolonged growth phase (Figs. 2C, D and 3B, E). Surprisingly, cell divisions were also maintained in the cauline leaf during the transition through its phase of slow growth between 4 and 7 DAI (Figs. 2C, D and 3B, E). Cell divisions and differentiation are intricately linked, and a delay in differentiation often leads to prolonged proliferative activity (Vuolo et al. 2018; Werner et al. 2021; Wu et al. 2021; Gómez-Felipe et al. 2024; Li et al. 2024). This trait of the maintenance of cell division may suggest that cells in the cauline leaf differentiate later than those in sepals and rosette leaves.

### Cell differentiation is delayed in the cauline leaf

**Figure 4.**
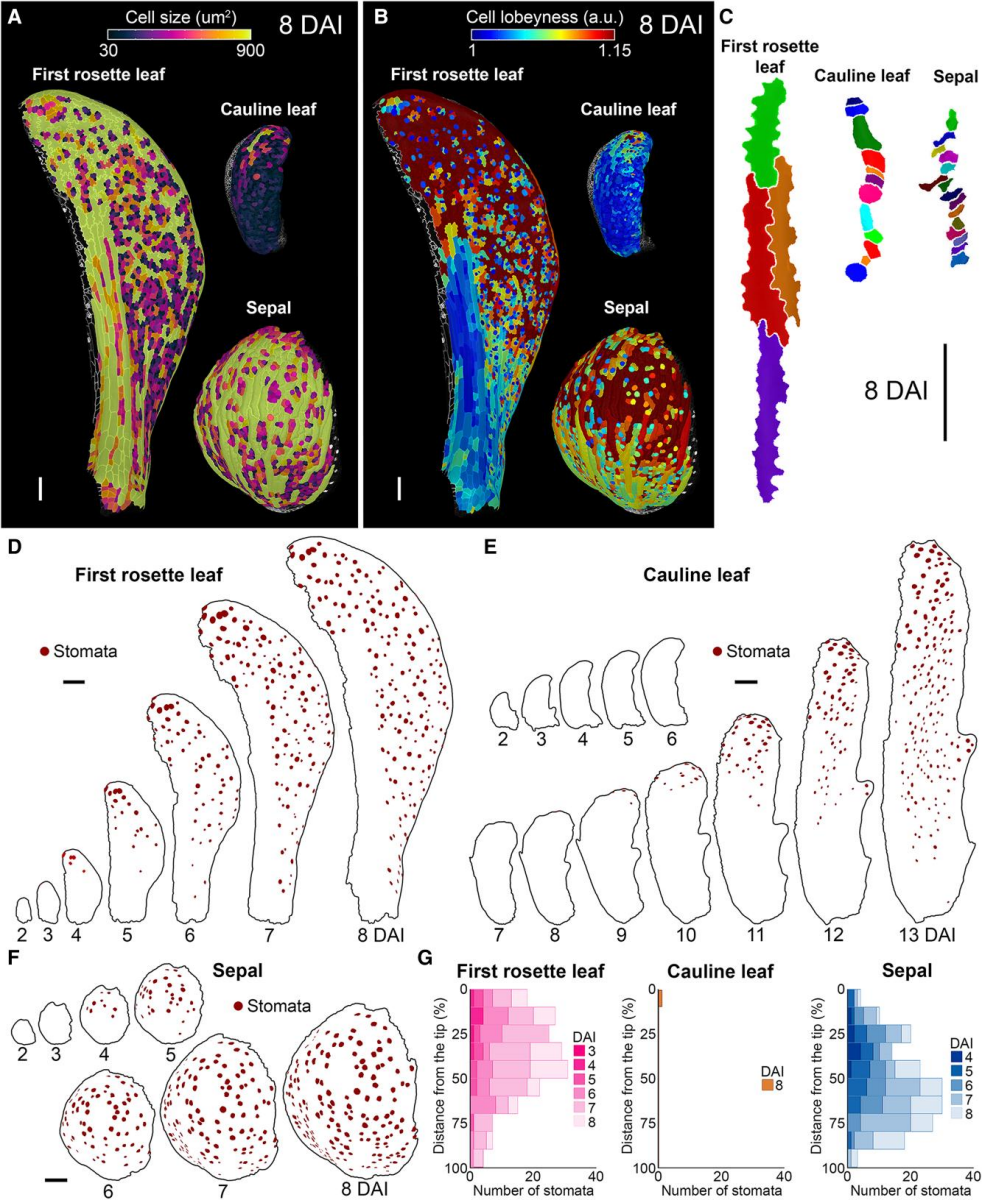
Cell differentiation is delayed in the cauline leaf. **A)** Heat maps of cell size in the first rosette leaf (left), cauline leaf (top), and sepal (bottom) at 8 DAI. **B)** Heat maps of cell lobeyness in the first rosette leaf (left), cauline leaf (top), and sepal (bottom) at 8 DAI. Heat maps are displayed on the digitally extracted organ surface. **C)** Geometries of representative cells located at the distal margin of the first rosette leaf (left), cauline leaf (middle), and sepal (right) at 8 DAI. **D to F)** Stomata distribution in the first rosette leaf **D)**, cauline leaf **E)**, and sepal **F)**. Stomata marked in brown. **G)** Quantification of stomatal distribution as a function of the distance from the tip of the organ for the first rosette leaf (left), cauline leaf (middle), and sepal (right). Bar plots represent the number of stomata. (*n* = 1 to 189 stomata, based on the time-lapse series shown in **D** to **F**). DAI indicates days after primordium initiation. Scale bars: 100 *µ*m. See also Supplementary Fig. S3.

To verify if cell differentiation is indeed delayed in the cauline leaf, we measured morphological markers of epidermal cell differentiation status such as cell size, stomata distribution, and pavement cell lobeyness (a measure of cell shape complexity; Andriankaja et al. 2012; Rodriguez et al. 2014; Sapala et al. 2018; Le Gloanec et al. 2022). The abaxial epidermis of first rosette leaves and sepals at 8 DAI consisted of big cells, which often developed extensive lobeyness in the leaf blade and margin or distal region of the sepal (Fig. 4A to C; Supplementary Fig. S3). At this stage, stomata were already present all over the blade of the first rosette leaf and the entire abaxial epidermis of the sepal (Fig. 4D, F, and G). By contrast, the cauline leaf epidermis, including the leaf margin, was mostly composed of small isodiametric cells. Only a few cells located at the tip of the first rosette leaf started to increase their size and became lobey (Fig. 4A to C; Supplementary Fig. S3). Occasionally, some individual stomata could be found in the distal region (Fig. 4E and G), indicating that, at this stage, the cauline leaf is mostly undifferentiated and non-photosynthetic. Stomata spread basipetally through the epidermis only at later stages (from 10 to 13 DAI), suggesting a delay in the establishment of photosynthetic activity in this organ (Fig. 4E). Thus, organ differentiation progresses much faster in both the first rosette leaf and sepal while it is markedly delayed in the cauline leaf.

### Growth is redistributed in the cauline leaf

During early development, cauline leaves display a strong delay in cell differentiation compared with the sepals and rosette leaves of the same size (Fig. 4 and Supplementary Fig. S3). This delay may lead to an increased contribution of the cells localized at the tip of the early primordium to the final surface of the cauline leaf. Such redistribution of growth, caused by the delay of organ differentiation, has been shown to underlie the development of leaf complexity in Brassicaceae (Kierzkowski et al. 2019).

**Figure 5.**
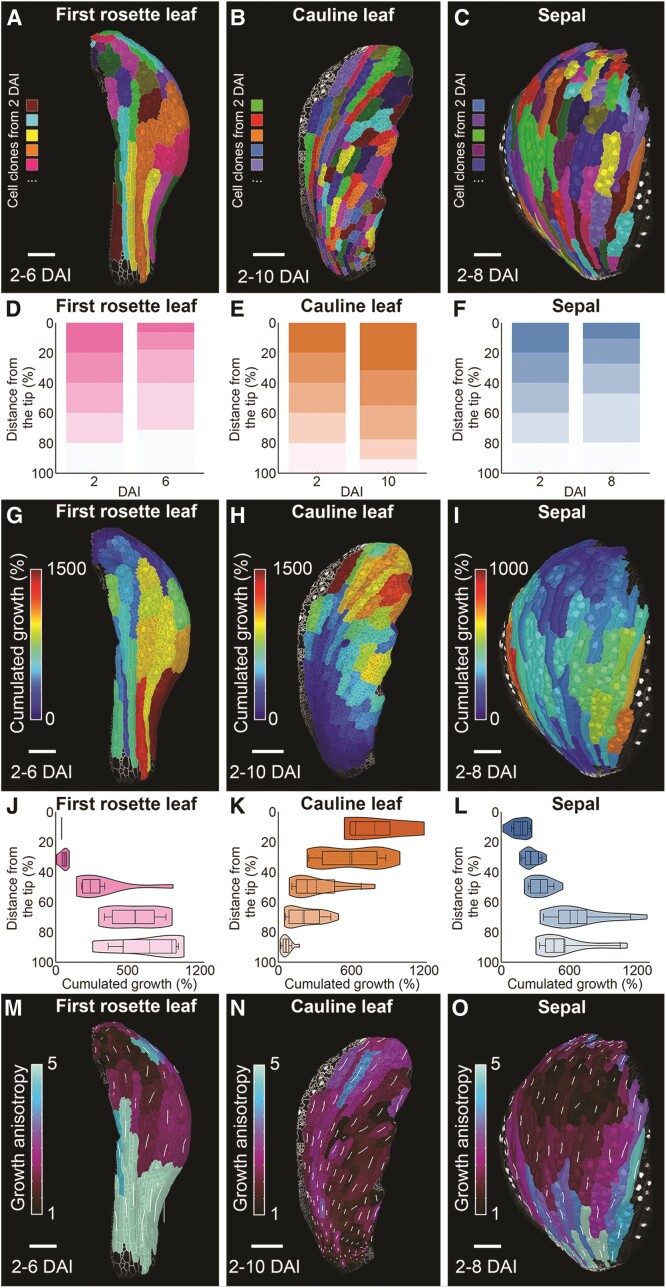
Growth is redistributed distally in the cauline leaf. **A to C)** Cell lineage tracing from 2 DAI to the indicated time point (*n* DAI) in the first rosette leaf **A)**, cauline leaf **B)**, and sepal **C)** of comparable size. Colors indicate clones developing from single cells at 2 DAI displayed on the digitally extracted organ surface at the indicated time point. **D to F)** Quantification of the contribution of the clones to the length of the organ at n DAI in the first rosette leaf **D)**, cauline leaf **E)**, and sepal **F)** of comparable size. **G to I)** Heat maps of cumulative area expansion (from 2 to n DAI) in the first rosette leaf **G)**, cauline leaf **H)**, and sepal **I)**. Heat maps generated between the two indicated time points are displayed on the digitally extracted organ surface at the later time point. **J to L)** Quantification of the cumulative area expansion (from 2 to n DAI) in the first rosette leaf **J)**, cauline leaf **K)**, and sepal **L)**. **M to O)** Heat maps of growth anisotropy in the first rosette leaf **M)**, cauline leaf **N)**, and sepal **O)** between 2 and n DAI (comparable size). Heat maps generated between the two indicated time points are displayed on the digitally extracted organ surface at the later time point. Stacked histogram represents the relative contribution of equal segments at 2 DAI to the organ length at n DAI in the samples show in **A to C**. Violin plots and boxplots represent 90% of the values; mean is indicated by a dashed line, median by a line, boxes represent interquartile ranges, and whiskers 95% of data (*n* = 3 to 7 clones, based on the sample shown in **G to I**). DAI indicates days after primordium initiation. Scale bars: 100 *µ*m. See also Supplementary Fig. S4.

To evaluate this possibility, we computed epidermal clonal lineages that developed from cells in early primordia (2 DAI) until all organs reached comparable sizes of around 700 *µ*m (Fig. 5A to C; Supplementary Fig. S4). Already at this early developmental period, the first rosette leaf displayed smaller clones at the tip, with the marginal cells often stemming from a single cell. By contrast, the basal area grew very fast, producing elongated sectors contributing to over 35% of the organ length at the comparable size of around 700 *µ*m (Fig. 5D, G, and J; Supplementary Fig. S4A, B, and G). Conversely, cells located at the distal region of the early primordium of the cauline leaf grew the fastest and increased their size substantially compared with those at the base (Fig. 5E, H, and K; Supplementary S4B and E). The contribution of the distal 20% of the primordium surface at 2 DAI increased to around 35% of the total organ surface at 10 DAI (Fig. 5E; Supplementary Fig. S4H). In this respect, the sepal resembled the first rosette leaf, showcasing a greater contribution from basal cells and smaller clones at the tip (Fig. 5F, I, and L; Supplementary Fig. S4C, F, and I).

Interestingly, the growth anisotropy of the clones in the upper half of the first rosette leaf tended to converge toward its tip (Fig. 5M). On the other hand, sectors in the cauline leaf tended to diverge from the main axis of the organ, pointing toward the distal edges of the leaf (Fig. 5N). To a lesser extent, we also observed divergent growth polarities in the sepal, but these sectors were smaller than those in the cauline leaf as they differentiated and ceased their growth early (Fig. 5M). This divergent growth anisotropy of clones in the cauline leaf may result from the maintained morphogenetic activity of its non-differentiated margin, which could act to reorient growth, as previously observed in the petal (Lampugnani et al. 2013; Sauret-Güeto et al. 2013).

### Auxin patterning mirrors the differential development of the cauline leaf

**Figure 6.**
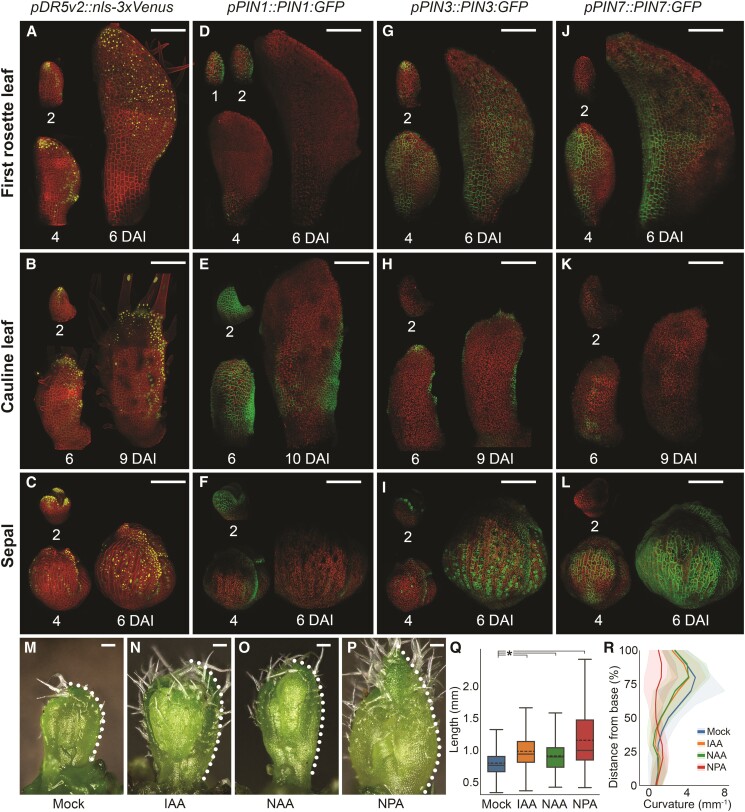
Auxin patterning mirrors the differential development of the cauline leaf. **A to C)** Expression patterns of *pDR5v2::nls-3xVenus* in the first rosette leaf **A)**, cauline leaf **B)**, and sepal **C)**. **D to F)** Expression patterns of *pPIN1::PIN1-GFP* in the first rosette leaf **D)**, cauline leaf **E)**, and sepal **F)**. **G and H)** Expression patterns of *pPIN3::PIN3-GFP* in the first rosette leaf **G)**, cauline leaf **H)**, and sepal **I)**. **J to L)** Expression patterns of *pPIN7::PIN7-GFP* in the first rosette leaf **J)**, cauline leaf **K)**, and sepal **L)**. Autofluorescence and TDT in red; Venus in yellow; GFP in green. Representative samples are shown (min. 3 replicates for each time point). DAI indicates days after primordium initiation. **M to P)** Representative morphologies of approximatively 9/10-day-old cauline leaves treated for 7 d with mock **M)**, 10 *µ*M Indole-3-acetic acid (IAA) **N)**, 10 *µ*M Naphthalene acetic acid (NAA), **O)**, or 10 *µ*M N-1-naphthylphthalamic acid (NPA) **P)** solutions. Dotted lines show the profile of each leaf along its abaxial epidermis. **Q)** Quantification of the cauline leaf length attained after chemical treatments. Boxplots represent 99% of the values; dashed lines represent mean, lines median, box limits represent interquartile range, and whiskers contain 99th percentile of data (*n* = 97 leaves for mock, 110 for IAA, 90 for NAA, and 87 for NPA treatments). Significant differences are marked by star with *P* < 0.005. **R)** Curvature of the cauline leaves along the normalized leaf length for different treatments. Line represents the median curvature for each percentile, and shadow SD. DAI indicates days after primordium initiation. Scale bars: 100 *µ*m.

Auxin is a key regulator of cell growth and differentiation (Benková et al. 2003; Ding and Friml 2010; Di Mambro et al. 2017; Kierzkowski et al. 2019). For instance, auxin maxima at the margin of the leaf and petal are known to locally coordinate cellular growth rates and anisotropy (Barkoulas et al. 2008; Sauret-Güeto et al. 2013; Fox et al. 2018; Kierzkowski et al. 2019; Zhang et al. 2020). To investigate if auxin patterning could underlie the differential growth observed in cauline leaves, we first monitored auxin responsiveness during organ development using the *DR5v2* reporter (Liao et al. 2015). At early stages (∼2 DAI), epidermal *DR5v2* signal was detected mainly at the tip of the first rosette leaf, and this localization was largely maintained at 4 DAI (Fig. 6A). At later stages (∼6 DAI), the *DR5v2* signal started to expand basally throughout the abaxial epidermis of the first rosette leaf (Fig. 6A). Interestingly, after the initial tip localization at 2 DAI, *DR5v2* signal extended along the distal edges of the cauline leaf but did not spread throughout the leaf blade at later stages (Fig. 6B). In contrast to both types of leaves, *DR5v2* domain was broader in the distal margin of the sepal from its initiation (Fig. 6C). Like in the cauline leaf, auxin responsiveness was strong in the sepal margin but also spread through the sepal epidermis as in the first rosette leaf (Fig. 6C). A broad marginal distribution of the DR5v2 signal along the margins correlated with divergent growth anisotropy of the clones in both sepals and cauline leaves (Fig. 5). This suggests that auxin accumulation in this domain could locally reorient the growth away from the organ tip as previously observed in petals (Lampugnani et al. 2013; Sauret-Güeto et al. 2013). However, this effect was weaker in sepal as its tip quickly differentiated and stopped growing (Figs. 2B and 4A to C, F, and G).

Auxin distribution throughout the tissue is mainly regulated by auxin efflux carriers from the *PINFORMED* (*PIN*) family. Therefore, we next monitored the distribution patterns of the *GFP* fusion lines of *PIN1*, *PIN3*, and *PIN7*, which are known to be involved in leaf development (Barkoulas et al. 2008; Guenot et al. 2012; Abley et al. 2016; Mansfield et al. 2018). At early stages (1-2 DAI), *PIN1* was present throughout the epidermis of all three organs (Fig. 6D to F). This *PIN1* expression was quickly eliminated from the epidermis of the first rosette leaf at around 4 DAI and was restricted to the organ margin in the sepal (Fig. 6D and F). Interestingly, *PIN1* signal persisted throughout abaxial epidermis of the cauline leaf until at least 6 DAI and later was still present in the proximal and lateral regions of the leaf coinciding with the initiation of the marginal serrations (Fig. 6E).

*PIN3* was expressed at the tip of the first rosette leaf and sepal from 2 DAI, while it was absent at this stage in the cauline leaf (Fig. 6G to I). The *PIN3* expression domain quickly (from 4 DAI) expanded basally through the epidermis of the first rosette leaf, while it was restricted to the margin of the cauline leaf (Fig. 6G and H). The sepal displayed an intermediate *PIN3* expression pattern: first being restricted to the organ margin at 4 DAI, then later expanding throughout the abaxial epidermis at 6 DAI (Fig. 6I). Finally, *PIN7* expression was absent at 2 DAI in all organs, and then quickly expanded through the epidermis of the first rosette leaf and sepal while it was only briefly detected in the medial and proximal regions of the cauline leaf at around 6 DAI (Fig. 6J to L). Altogether, we found striking differences in auxin patterning that could underlie the differential growth and differentiation patterns between these three laminar organs.

To test if the delay in auxin response in the leaf blade could underlie the specific developmental trajectory of cauline leaves, we sprayed their initiating primordia with exogenous auxins. Both indole-3 acetic acid (IAA) and naphthalene acetic acid (NAA) treatments accelerated the leaf development leading to the formation of significantly longer and less curled cauline leaves as compared with the mock treatment (Fig. 6M to O and Q, R). This is consistent with previous results, where auxin treatment accelerated both cellular growth and differentiation in the adult leaf (Kierzkowski et al. 2019). This suggests that the delay in auxin spread through the cauline leaf may be the underlying factor in their slow initial growth.

Prolonged expression of *PIN1* in the blade of the cauline leaf (Fig. 6E) could prevent early auxin accumulation in its abaxial epidermis (Fig. 6B), leading to slower growth and delaying differentiation in this organ as compared with rosette leaves. To further test this idea, we blocked the activity of *PIN* transporters using N-1-naphthylphthalamic acid (NPA) treatment during the early development of cauline leaves. Like auxin treatment, NPA also accelerated early leaf development, resulting in much larger and straighter cauline leaves as compared with the mock treatment (Fig. 6M, P and M to R). Altogether, these results suggest that PIN-mediated restriction of auxin spread through the organ epidermis may be the underlying cause of the early developmental delay observed in cauline leaves.

## Discussion

**Figure 7.**
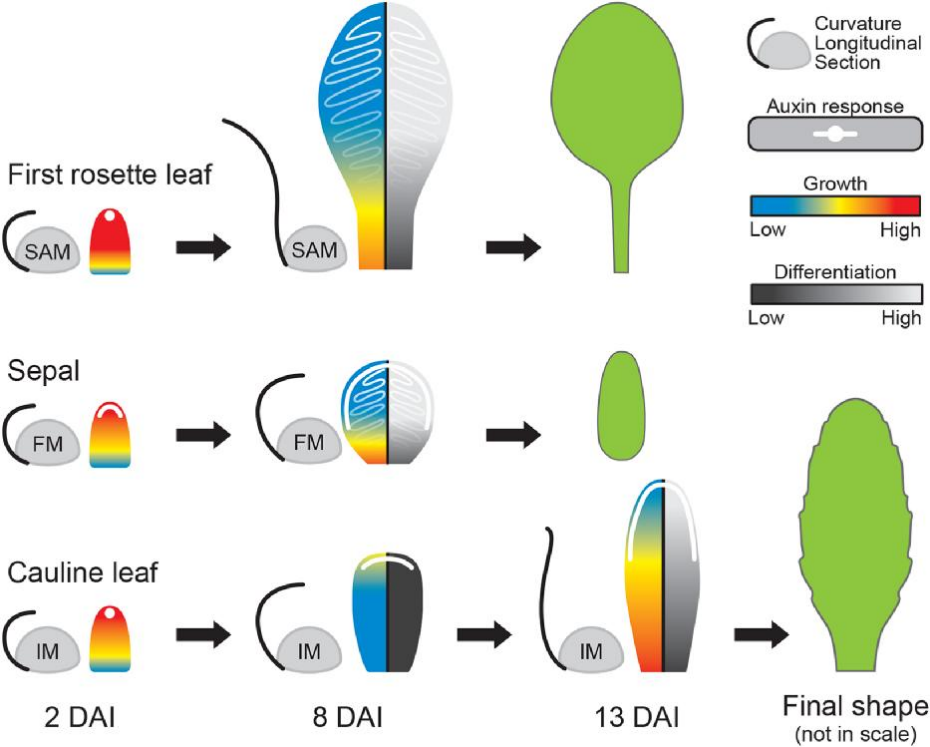
Quantitative modulation of growth and differentiation underlies cauline leaf development. Schematic representation of *A*. *thaliana* first rosette leaf, cauline leaf, and sepal shapes. Growth rates are depicted through blue-red gradients and differentiation rates by black-gray gradients, while the white color indicates the temporal distribution of auxin at specific developmental time points. All organs initiate as finger-shaped, fast-growing primordia that are curled toward meristems. In the first rosette leaf, growth and differentiation gradients are rapidly established, accompanied by auxin redistribution throughout the epidermis, and the leaf unfolds quickly. Growth and differentiation gradients in the sepal also rapidly progress; auxin is redistributed throughout the leaf margin and the epidermis, and the organ remains curled toward the flower. In contrast, the cauline leaf undergoes a transient slow-growing phase when cells remain undifferentiated, and auxin is retained at its distal margin. During this slow-growing phase, the cauline leaf stays curled toward the inflorescence. At late stages, growth rates increase again, gradients of growth and differentiation start to progress and the leaf unfolds. DAI indicates days after primordium initiation.

In this study, we performed a detailed cell-level analysis of development in the cauline leaf and uncovered what sets it apart from other aerial organs, such as rosette leaves and sepals. We revealed that two main factors drive its functional distinction: (1) the timing of cell differentiation and (2) the speed and distribution of cellular growth. More specifically, cauline leaves experience a strong delay in cell maturation (Figs. 3 and 7). Consequently, they display an extended period of proliferative activity and a global redistribution in growth toward the more distal parts of the organ (Figs. 2, 4, 5 and 7). Remarkably, cauline leaves undergo a transient phase of slow expansion followed by a resumption of rapid growth (Figs. 1 and 2).

Tuning cell differentiation is known to be critical for shaping plant organs (Efroni et al. 2010; Rodriguez et al. 2014; Alvarez et al. 2016). For example, delaying the transition from cell proliferation to cell maturation by class I Knotted-like Homeobox (*KNOXI*) genes has been shown to be a key component allowing the development of complex leaf shape (Hay and Tsiantis 2006; Barth et al. 2009; Nakayama et al. 2014; Kierzkowski et al. 2019; Wang et al. 2022). Interestingly, the expression of *KNOXI* genes leads to a redistribution of growth within the organ, resulting in an increased contribution of distal organ regions to the final organ shapes (Kierzkowski et al. 2019). A similar mechanism has been observed in floral organs such as petals, where a broad distal region develops only when cell differentiation is suppressed by the action of *JAGGED* (*JAG*), which downregulates cell cycle inhibitors (Schiessl et al. 2012). More broadly, the miR396-GEF module controls cell proliferation and differentiation, regulating growth redistribution in eudicot species (Das Gupta and Nath 2015). The delay of cell differentiation in the cauline leaf also correlates with a greater contribution of its distal region to the organ surface (Fig. 5), supporting the idea that delaying cell differentiation may be a general mechanism for how plant organs change their proportions.

Interestingly, the transition from juvenile to adult phases in *Arabidopsis* leaves controlled by SQUAMOSA-promoter binding protein-like (*SPL*) genes has also been found to influence leaf morphology by delaying maturation while promoting growth and cell divisions in an age-dependent manner (Schwarz et al. 2008; Xu et al. 2016; He et al. 2018; Tang et al. 2023; Li et al. 2024). *SPL9* and *SPL10* have been shown to target genes involved in cell cycle regulation, such as *CYCLIN D3;3,* and *A2;3* (Tang et al. 2023; Li et al. 2024). As the cauline leaf marks the extremity of the heteroblastic series, the regulation of *SPL*s may underlie the strong delay in cell maturation observed in this organ. Indeed, repression of *SPL10* genes results in noticeable morphological changes in the cauline leaf, converting its shape toward the rosette leaf, including the development of a broader leaf blade (Shikata et al. 2009; Huijser and Schmid 2011).

Auxin is known to accelerate cell differentiation and growth in *Arabidopsis* rosette leaves (Challa et al. 2019; Kierzkowski et al. 2019; Zhang et al. 2020). Here, we found that the progression of the overall cell differentiation correlates with a basipetal spread of auxin response through the abaxial epidermis and that this progression was delayed in the cauline leaf as compared with the sepal and first rosette leaf (Fig. 6). Supplying external auxin led to faster cauline leaf growth (Fig. 6) suggesting that restriction of auxin spread through the cauline leaf may be the underlying cause of its early developmental delay.

Early auxin response detected in the blade of the first rosette leaf and sepal correlated with the elimination of the *PIN1* auxin efflux carrier from the abaxial epidermis that was quickly replaced by the basally progressing expression of *PIN3* and *PIN7* transporters (Fig. 6). By contrast, we observed a prolonged expression of *PIN1*, and the corresponding restriction of auxin response to the margin of the cauline leaf (Fig. 6). As in the case of auxin treatment, blocking the activity of auxin efflux carriers at early developmental stages in the cauline leaf led to faster growth (Fig. 6) suggesting that *PIN1* may be restricting auxin to the leaf margin in this organ. This data is consistent with recent results from the gynoecium where changes in *PIN*-mediated polar auxin transport were tightly correlated with the onset of cell differentiation in the style (Gómez-Felipe et al. 2024). Thus, modulation of active polar auxin transport may be broadly involved in controlling the basipetal progression of cell differentiation through lateral organs in plants.

Our findings indicate that marginal cells in rosette leaves undergo early differentiation, while in the cauline leaf, cells remain smaller and mainly undifferentiated (Fig. 4). *PIN3* expression expanded during the early stages of the cauline leaf development and coincided with broad redistribution of *DR5v2* signal through the distal margin (Fig. 6). Such *PIN3*-mediated auxin redistribution at the margin has been suggested to coordinate growth orientations in the petal, leading to the creation of the divergent growth polarity in its distal end (Lampugnani et al. 2013; Sauret-Güeto et al. 2013). A similar mechanism seems to operate during the early development of the cauline leaf, where growth in the distal blade is oriented toward its margin (Fig. 5). However, such reorientation of growth in the cauline leaf and petal may occur only because of their general delay in cell differentiation in the epidermis. In sepals, despite the broad distribution of *PIN3*, higher accumulation of *DR5v2* signal, and smaller cells at the margins, the early onset of basipetal gradient of differentiation in the epidermis quickly reduces the ability to reorient growth toward the margin.

Interestingly, both sepals and cauline leaves are bent toward the inside of the flower for a prolonged period and do not quickly unfold like rosette leaves (Fig. 1) (Smyth et al. 1990; Pabõn-Mora et al. 2013). The delay of differentiation combined with the distally localized growth in the early cauline leaf may help to prevent its early unfolding. While the mechanism underlying this change in curvature remains unclear, current evidence suggests that it likely involves differential growth between both organ surfaces (Zhao et al. 2020; Jiao et al. 2021; Yadav et al. 2023). Such growth asymmetry might be controlled by differential gene expression during the establishment of organ polarity. For instance*, AUXIN RESPONSE FACTOR 3* (*ARF3*) also named *ETTIN* (*ETT*) marks the abaxial side of the incipient leaf primordia and was shown to regulate the elongation of the valve tissue in the gynecium (Andres-Robin et al. 2018; Burian et al. 2022). Although the growth dynamics observed here in the abaxial epidermis clearly highlight major developmental differences between laminar organs, future studies should focus on further quantifying development in different organ layers, including the adaxial epidermis, to uncover how laminar organ geometries and curvatures are tuned in space and time.

In contrast to rosette leaves, which exhibit a sigmoid growth curve influenced by leaf age (Cookson and Granier 2006; Baerenfaller et al. 2015), cauline leaves display a distinct pattern: an initial rapid decline in growth rates, followed by a surprising growth resumption (Figs. 1 and 2). Although the genetically induced delay of cell differentiation is known to cause a decrease in growth rates (Kierzkowski et al. 2019; Wang et al. 2022), the temporary slowdown in cell expansion followed by an acceleration of growth has never been documented during normal development of *Arabidopsis* leaves. However, such growth dynamics is characteristic for some floral organs, such as petals and filaments of the stamen (Smyth et al. 1990; Sauret-Güeto et al. 2013; Silveira et al. 2022). The genetic factors driving late-stage acceleration in growth remain unclear but phytohormones such as auxin, jasmonate, and gibberellins are believed to be key players, especially in the growth resumption of the stamen (Nagpal et al. 2005; Reeves et al. 2012; Cecchetti et al. 2013; Huang et al. 2020; He et al. 2023). Given the genetic proximity between cauline leaves and floral organs (Krizek and Meyerowitz 1996; Pelaz et al. 2001), it would be interesting to investigate whether similar mechanisms govern cauline leaf development to help fulfill its dual function.

Finally, growth cessation also occurs during bud dormancy when the shoot apex inhibits axillary bud development—a phenomenon known as apical dominance (Junttila and Hänninen 2012; Bredmose and Costes 2017). Cauline leaves are initiated before the onset of the dormancy of the axillary buds, thus molecular signals controlling apical dominance and bud dormancy may also be involved in the transient partial inhibition of growth and subsequent growth resumption in these leaves (Rinne et al. 2011; Cooke et al. 2012; Liu et al. 2016; Salem et al. 2018; Hao et al. 2019; Martignago et al. 2020).

Our quantitative study brings insights into the crucial role of temporal dynamics in orchestrating proper morphogenesis and functional maturation in laminar organs in plants. It provides a solid framework to investigate the molecular mechanisms underlying the development of the cauline leaf, enabling its transition from protection to photosynthesis. Such an approach could offer a deeper understanding of how these organs, originating from a common ancestor, have evolved to fulfill distinct functional roles.

## Materials and methods

### Plant material and growth conditions

The following Arabidopsis (*A. thaliana*) transgenic lines were used in this study: *pUBQ10::myr-YFP* (Willis et al. 2016), *pUBQ10::PM-TDT* (Melnyk et al. 2015), *pDR5v2::nls-3xVenus* (Liao et al. 2015), *pPIN1::PIN1-GFP* (Kierzkowski et al. 2013), *pPIN3::PIN3-GFP* (Žádníkova et al. 2010), and *pPIN7::PIN7-GFP* (Belteton et al. 2018). All lines are in the Col-0 background except for the DR5 reporter line, which is in the Columbia/Utrecht background. *pDR5v2::nls-3xVenus* was crossed with *pUBQ10::PM-TDT* and observed in the 5th generation. Seeds were stratified in the dark for two days at 4°C to synchronize germination. Plants were grown on soil in a growth chamber under long-day conditions (16 h/8 h light/dark period, 95 *µ*mol m^−2^ s^−1^) at 22 ± 1°C with 60% to 70% relative humidity.

### Chemical treatments

Thirteen-day-old Col-0 plants (grown on soil under long-day conditions as described above) were sprayed with 10 *µ*M NAA, 10 *µ*M N-1- NPA, or 10 *µ*M IAA dissolved in water with 0.01% (v/v) Silwet-77, or mock solution, once time per day for seven consecutive days. Solutions were sprayed on the apex of each plant, with each plant receiving approximatively 0.7 to 0.8 ml of solution per day.

### Organ-scale measurements

Plants cultivated under the above-described conditions were standardized by selecting individuals of comparable sizes. Organs at consecutive developmental stages were imaged with one-day intervals from 2 to 19-day-old plants for first rosette leaves, from 10 to 30-day-old plants for cauline leaves (up to two oldest and clearly distinguishable cauline leaves per sample—from first to third), and from 20 to 33-day-old-plant for abaxial sepals. All images were acquired using a Keyence digital microscope model VHX-970F. The lengths of all organs were measured using FIJI following the abaxial surface from the base to the tip of the organ (Schinidelin et al. 2012).

For chemical treatments, the plants were dissected 7 to 8 d after the initial treatment (20 to 21 d old plants). All rosette leaves were removed, and the cauline leaves were imaged with the Keyence microscope. One or two oldest cauline leaves per plant were measured using the Segmented Line tool in FIJI (Schinidelin et al. 2012), by drawing a line from the base to the tip, following the curvature along the midvein on the leaf profile. The coordinates of this line were used to generate splines from which the curvature along the length was calculated. Curvature, *κ*, for a function y=f(x) is defined as $\kappa =\frac{x^{\prime \prime }y^{\prime }-x^{\prime }y^{\prime \prime }}{{\left({x^{\prime }}^{2}+{y^{\prime }}^{2}\right)}^{3/2}}$, where ${\mathrm{prime}}^{\prime }$ indicates a derivative. Coordinates of generated splines were applied to this equation using the *gradient* function from Numpy to obtain curvatures and were normalized over leaf length.

### Analysis of organ-scale growth

Measurements of organ length from the developing first rosette leaves, cauline leaves, and sepals were fitted to an analytical function L(t). A sigmoid function $L(t)=\frac{a}{1+e^{\left(-c(t-b)\right)}}+d$, where *t* is the time in days, was fitted to the measured length data from the rosette leaf, its blade and petiole (Fig. 1D; Supplementary Fig. S1) using *optimize.curve_fit* from SciPy. The parameters in this sigmoid function describe the curve shape—*a* is the maximum value of the function, *b* is the *t* value of the function's midpoint, *c* is the steepness of the curve at its midpoint, and *d* is a constant offset. Splines were used to fit the length of the cauline leaf and sepal using *interpolate.BSpline* and *interpolate.splerep* with smoothing condition s=0.1 from SciPy. AGR, defined as the first derivative over time of the fitted function L(t), was calculated using the *gradient* function in NumPy (Fig. 1E). RGR in percent per day was defined as $R(t)=100\;e^{\left(r(t)-1\right)}$, where $r(t)=\frac{\ln\left(\frac{L(t)}{L(t-\Delta t)}\right)}{\Delta t}$, and was calculated from the fitted L(t) function (Fig. 1F).

### Confocal time-lapse imaging

The *pUBQ10::myr-YFP* transgenic line was used in all time-lapse experiments to visualize cell outlines and quantify cell expansion, cell proliferation, cell differentiation, and cell lineages.

For cauline leaf, cotyledons and older leaf primordia were removed using fine tweezers or an injection needle from two-week-old plants to expose the initiating third cauline leaf primordium. The dissected plants were placed horizontally in Ø60 mm Petri dishes filled with ½ Murashige and Skoog (MS) medium, supplemented with 1.5% agar, 1% sucrose, and 0.1% Plant Protective Mixture (Plant Cell Technology). Plants were immersed in water containing 0.1% PPM for imaging. For each replicate, at least half of the abaxial leaf surface was imaged at 24 h intervals for up to 10 d. Between imaging, water was removed, and samples were cultured in vitro under standard long-day conditions in a growth chamber. The images shown in Figs. 2A, 3A, and 4D and Supplementary Figs. S2A, D, and S3A, D are derived from two independent overlapping series (2 to 7 DAI and 8 to 13 DAI). Time-lapse imaging of juvenile leaves and sepals was previously described (Hervieux et al. 2016; Le Gloanec et al. 2022).

All confocal imaging was performed with an LSM800 upright confocal microscope using a long-distance water-dipping objective (AP 40X/1.0; Zeiss). Excitation was performed using a diode laser with 488 nm for YFP, GFP, Venus, and 561 nm for TDT and autofluorescence. Images were collected at 500 to 550 nm for YFP, GFP, and Venus, 560 to 650 nm for TDT, and 600 to 700 nm for autofluorescence. Confocal stacks were acquired at 512 × 512 resolution and 16-bit image depth, with 0.5 to 2 *µ*m distance in *Z*-dimension. Imaging was performed with the same confocal settings for each fluorescence marker line. For samples larger than the field of view, multiple overlapping stacks were obtained and later stitched together using MorphoGraphX (Barbier de Reuille et al. 2015; Strauss et al. 2022).

### Confocal image analysis

Cellular growth and expression quantifications were conducted using MorphoGraphX. Stacks were processed as described previously (Le Gloanec et al. 2022). After digitally removing the trichomes when necessary, the organ surface was detected with the “Edge detect” tool with a threshold of 6,000 to 13,000, followed by the “Edge detect angle” (3,000 to 6,500). An initial 5 *µ*m cube size mesh was then created and subdivided three times before projecting the membrane signal (2 to 5 *µ*m).

Cell segmentation and parent attribution were performed manually. Verification of cell parenting was done using “Check correspondence”. Lineages over multiple days were calculated as described previously and manually corrected when required (Kierzkowski et al. 2019). Stomata were identified manually based on their morphology and developmental trajectory (Le Gloanec et al. 2022).

Metrics such as area expansion, cell proliferation, growth anisotropy, lobeyness, and cell size were computed as described before (Barbier de Reuille et al. 2015; Sapala et al. 2018). Briefly, to compute growth, we compared the surface area of the mother cell at a given time point with the surface area of all its daughter cells at the next time point. Cell proliferation was computed by counting the number of daughter cells originating from the mother cell at an earlier time point. Growth rates along proximodistal and mediolateral axes were determined using a custom Bezier grid, manually adjusted to follow the organ geometry at each time point closely. Proximodistal and mediolateral distances from the organ base were calculated using the “Cell distance” plugin, employing the “Euclidean” parameter.

All figures were assembled using Adobe Photoshop or Adobe Illustrator software.

### Quantification and statistical analysis

Python scripts were used to generate plots in Figs. 1D, 2D, F, H, 3, 5, and 6 and Supplementary Figs. 2 and 3, while R scripts were used for Figs. 2E, G, I, and 4. Violin plots in Figs. 2D and 5, J to L represent value distributions, encompassing 90% of the data. Ribbon plots in Fig. 2E indicate the mean with shaded areas representing standard deviations. Stacked histograms in Fig. 5D assess the relative contribution of equal segments at 2 DAI during specific time points. Bar plots in Fig. 3D to F represent the relative proportions of each value per time point. In Fig. 6R, median and standard deviations were taken at each normalized length value (0 to 1 in increments of 0.1) and plotted using the *plot* function from Matplotlib. Statistical significance was determined using a two-sample *t*-test, with signifying *P*-value of *P* < 0.005.

## Supplementary Material

kiae408_Supplementary_Data

## Data Availability

Raw data and all scripts used to analyze data are available to download from the Open Science Framework repository: https://osf.io/uth78/.

## Supplementary data

The following materials are available in the online version of this article.

**Supplementary Figure S1.** Growth dynamics of the first rosette leaf regions.

**Supplementary Figure S2.** The establishment of the proximodistal growth gradient is delayed in the cauline leaf.

**Supplementary Figure S3.** Cauline leaf differentiates at later stages.

**Supplementary Figure S4.** Growth is redistributed distally in the cauline leaf.

## References

[kiae408-B1] Abley K , Sauret-Güeto S, Marée AFM, Coen E. Formation of polarity convergences underlying shoot outgrowths. Elife. 2016:5:e18165. 10.7554/eLife.18165 27478985 PMC4969039

[kiae408-B2] Alvarez JP , Furumizu C, Efroni I, Eshed Y, Bowman JL. Active suppression of a leaf meristem orchestrates determinate leaf growth. Elife. 2016:5:e15023. 10.7554/eLife.15023 27710768 PMC5096885

[kiae408-B3] Andres-Robin A , Reymond MC, Dupire A, Battu V, Dubrulle N, Mouille G, Lefebvre V, Pelloux J, Boudaoud A, Traas J, et al Evidence for the regulation of gynoecium morphogenesis by *ETTIN* via cell wall dynamics. Plant Physiol. 2018:178(3):1222–1232. 10.1104/pp.18.00745 30237208 PMC6236608

[kiae408-B4] Andriankaja M , Dhondt S, DeBodt S, Vanhaeren H, Coppens F, DeMilde L, Mühlenbock P, Skirycz A, Gonzalez N, Beemster GTS, et al Exit from proliferation during leaf development in *Arabidopsis thaliana*: a not-so-gradual process. Dev Cell. 2012:22(1):64–78. 10.1016/j.devcel.2011.11.011 22227310

[kiae408-B5] Baerenfaller K , Massonnet C, Hennig L, Russenberger D, Sulpice R, Walsh S, Stitt M, Granier C, Gruissem W. A long photoperiod relaxes energy management in *Arabidopsis* leaf six. Curr Plant Biol. 2015:2:34–45. 10.1016/j.cpb.2015.07.001

[kiae408-B6] Bar M , Ori N. Leaf development and morphogenesis. Development. 2014:141(22):4219–4230. 10.1242/dev.106195 25371359

[kiae408-B7] Barbier de Reuille P , Routier-Kierzkowska A-L, Kierzkowski D, Bassel GW, Schüpbach T, Tauriello G, Bajpai N, Strauss S, Weber A, Kiss A, et al MorphoGraphX: a platform for quantifying morphogenesis in 4D. Elife. 2015:4:05864. 10.7554/eLife.05864 25946108 PMC4421794

[kiae408-B8] Barkoulas M , Hay A, Kougioumoutzi E, Tsiantis M. A developmental framework for dissected leaf formation in the *Arabidopsis* relative *Cardamine hirsuta*. Nat Genet. 2008:40(9):1136–1141. 10.1038/ng.189 19165928

[kiae408-B9] Barth S , Geier T, Eimert K, Watillon B, Sangwan RS, Gleissberg S. KNOX overexpression in transgenic Kohleria (Gesneriaceae) prolongs the activity of proximal leaf blastozones and drastically alters segment fate. Planta. 2009:230(6):1081–1091. 10.1007/s00425-009-0997-0 19685246

[kiae408-B10] Belteton SA , Sawchuk MG, Donohoe BS, Scarpella E, Szymanski DB. Reassessing the roles of PIN proteins and anticlinal microtubules during pavement cell morphogenesis. Plant Physiol. 2018:176(1):432–449. 10.1104/pp.17.01554 29192026 PMC5761804

[kiae408-B11] Ben-Gera H , Dafna A, Alvarez JP, Bar M, Mauerer M, Ori N. Auxin-mediated lamina growth in tomato leaves is restricted by two parallel mechanisms. Plant J. 2016:86(6):443–457. 10.1111/tpj.13188 27121172

[kiae408-B12] Benková E , Michniewicz M, Sauer M, Teichmann T, Seifertová D, Jürgens G, Friml J. Local, efflux-dependent auxin gradients as a common module for plant organ formation. Cell. 2003:115(5):591–602. 10.1016/S0092-8674(03)00924-3 14651850

[kiae408-B13] Berhin A , Nawrath C, Hachez C. Subtle interplay between trichome development and cuticle formation in plants. New Phytol. 2021:233(5):2036–2046. 10.1111/nph.17827 34704619

[kiae408-B14] Bielczynski LW , Łaçki MK, Hoefnagels I, Gambin A, Croce R. Leaf and plant age affects photosynthetic performance and photoprotective capacity. Plant Physiol. 2017:175(4):1634–1648. 10.1104/PP.17.00904 29018097 PMC5717728

[kiae408-B15] Bilsborough GD , Runions A, Barkoulas M, Jenkins HW, Hasson A, Galinha C, Laufs P, Hay A, Prusinkiewicz P, Tsiantis M. Model for the regulation of *Arabidopsis thaliana* leaf margin development. Proc Natl Acad Sci U S A. 2011:108(8):3424–3429. 10.1073/pnas.1015162108 21300866 PMC3044365

[kiae408-B16] Bowman JL , Smyth DR, Meyerowitz EM. Genetic interactions among floral homeotic genes of *Arabidopsis*. Development. 1991:112(1):1–20. 10.1242/dev.112.1.1 1685111

[kiae408-B17] Bredmose N , Costes E. Axillary bud growth. In: Reference module in life sciences. 2017. 10.1016/B978-0-12-809633-8.05056-1

[kiae408-B18] Burian A , Paszkiewicz G, Nguyen KT, Meda S, Raczyńska-Szajgin M, Timmermans MCP. Specification of leaf dorsiventrality via a prepatterned binary readout of a uniform auxin input. Nat Plants. 2022:8(3):269–280. 10.1038/s41477-022-01111-3 35318449

[kiae408-B19] Burko Y , Ori N. The tomato leaf as a model system for organogenesis. In: De Smet I, editor. Methods in molecular biology. Totowa (NJ): Humana Press Inc; 2013. p. 1–19. 10.1007/978-1-62703-221-6_1 23299665

[kiae408-B20] Cecchetti V , Altamura MM, Brunetti P, Petrocelli V, Falasca G, Ljung K, Costantino P, Cardarelli M. Auxin controls *Arabidopsis* anther dehiscence by regulating endothecium lignification and jasmonic acid biosynthesis. Plant J. 2013:74(3):411–422. 10.1111/tpj.12130 23410518

[kiae408-B21] Challa KR , Rath M, Nath U. The CIN-TCP transcription factors promote commitment to differentiation in *Arabidopsis* leaf pavement cells via both auxin-dependent and independent pathways. PLOS Genet. 2019:15(2):e1007988. 10.1371/journal.pgen.1007988 30742619 PMC6386416

[kiae408-B22] Challa KR , Rath M, Sharma AN, Bajpai AK, Davuluri S, Acharya KK, Nath U. Active suppression of leaflet emergence as a mechanism of simple leaf development. Nat Plants. 2021:7(9):1264–1275. 10.1038/s41477-021-00965-3 34312497

[kiae408-B23] Cooke JEK , Eriksson ME, Junttila O. The dynamic nature of bud dormancy in trees: environmental control and molecular mechanisms. Plant Cell Environ. 2012:35(10):1707–1728. 10.1111/j.1365-3040.2012.02552.x 22670814

[kiae408-B24] Cookson SJ , Granier C. A dynamic analysis of the shade-induced plasticity in *Arabidopsis thaliana* rosette leaf development reveals new components of the shade-adaptative response. Ann Bot. 2006:97(3):443–452. 10.1093/aob/mcj047 16371443 PMC2803649

[kiae408-B25] Das Gupta M , Nath U. Divergence in patterns of leaf growth polarity is associated with the expression divergence of miR396. Plant Cell. 2015:27(10):2785–2799. 10.1105/tpc.15.00196 26410303 PMC4682314

[kiae408-B26] Di Mambro R , De Ruvo M, Pacifici E, Salvi E, Sozzani R, Benfey PN, Busch W, Novak O, Ljung K, Di Paola L, et al Auxin minimum triggers the developmental switch from cell division to cell differentiation in the *Arabidopsis* root. Proc Natl Acad Sci U S A. 2017:114(36):E7641–E7649. 10.1073/pnas.1705833114 28831001 PMC5594665

[kiae408-B28] Ding M , Zhu Y, Kinoshita T. Stomatal properties of *Arabidopsis* cauline and rice flag leaves and their contributions to seed production and grain yield. J Exp Bot. 2023:74(6):1957–1973. 10.1093/jxb/erac492 36520996 PMC10049919

[kiae408-B27] Ding Z , Friml J. Auxin regulates distal stem cell differentiation in *Arabidopsis* roots. Proc Natl Acad Sci U S A. 2010:107(26):12046–12051. 10.1073/pnas.1000672107 20543136 PMC2900669

[kiae408-B29] Donnelly PM , Bonetta D, Tsukaya H, Dengler RE, Dengler NG. Cell cycling and cell enlargement in developing leaves of *Arabidopsis*. Dev Biol. 1999:215(2):407–419. 10.1006/dbio.1999.9443 10545247

[kiae408-B30] Echevin E , Le Gloanec C, Skowrońska N, Routier-Kierzkowska A-L, Burian A, Kierzkowski D. Growth and biomechanics of shoot organs. J Exp Bot. 2019:70(14):3573–3585. 10.1093/jxb/erz205 31037307

[kiae408-B31] Efroni I , Eshed Y, Lifschitz E. Morphogenesis of simple and compound leaves: a critical review. Plant Cell. 2010:22(4):1019–1032. 10.1105/tpc.109.073601 20435903 PMC2879760

[kiae408-B32] Fox S , Southam P, Pantin F, Kennaway R, Robinson S, Castorina G, Sánchez-Corrales YE, Sablowski R, Chan J, et al Spatiotemporal coordination of cell division and growth during organ morphogenesis. PLoS Biol. 2018:16(11):e2005952. 10.1371/journal.pbio.2005952 30383040 PMC6211367

[kiae408-B33] Fukushima K , Hasebe M. Adaxial–abaxial polarity: the developmental basis of leaf shape diversity. Genesis. 2014:52(1):1–18. 10.1002/dvg.22728 24281766

[kiae408-B34] Gómez-Felipe A , Branchini E, Wang B, Marconi M, Bertrand-Rakusova H, Stan T, Burkiewicz J, de Folter S, Routier-Kierzkowska A-L, Wabnik K, et al Two orthogonal differentiation gradients locally coordinate fruit morphogenesis. Nat Commun. 2024:15(1):2912. 10.1038/s41467-024-47325-1 38575617 PMC10995178

[kiae408-B35] Green AA , Kennaway JR, Hanna AI, Bangham JA, Coen E. Genetic control of organ shape and tissue polarity. PLoS Biol. 2010:8(11):e1000537. 10.1371/journal.pbio.1000537 21085690 PMC2976718

[kiae408-B36] Guenot B , Bayer E, Kierzkowski D, Smith RS, Mandel T, Zádníková P, Benková E, Kuhlemeier C. PIN1-independent leaf initiation in *Arabidopsis*. Plant Physiol. 2012:159(4):1501–1510. 10.1104/pp.112.200402 22723086 PMC3425194

[kiae408-B37] Hamant O , Saunders TE. Shaping organs: shared structural principles across kingdoms. Annu Rev Cell Dev Biol. 2020:36(1):385–410. 10.1146/annurev-cellbio-012820-103850 32628862

[kiae408-B38] Hao X , Tang H, Wang B, Wang L, Cao H, Wang Y, Zeng J, Fang S, Chu J, Yang Y, et al Gene characterization and expression analysis reveal the importance of auxin signaling in bud dormancy regulation in tea plant. J Plant Growth Regul. 2019:38(1):225–240. 10.1007/s00344-018-9834-7

[kiae408-B39] Harline K , Roeder AHK. An optimized pipeline for live imaging whole *Arabidopsis* leaves at cellular resolution. Plant Methods. 2023:19(1):1–14. 10.1186/s13007-023-00987-2 36726130 PMC9890716

[kiae408-B40] Hay A , Tsiantis M. The genetic basis for differences in leaf form between *Arabidopsis thaliana* and its wild relative *Cardamine hirsuta*. Nat Genet. 2006:38(8):942–947. 10.1038/ng1835 16823378

[kiae408-B42] He J , Xu M, Willmann MR, McCormick K, Hu T, Yang L, Starker CG, Voytas DF, Meyers BC, Poethig RS. Threshold-dependent repression of SPL gene expression by miR156/miR157 controls vegetative phase change in *Arabidopsis thaliana*. PLOS Genet. 2018:14(4):e1007337. 10.1371/journal.pgen.1007337 29672610 PMC5929574

[kiae408-B41] He Y , He X, Wang X, Hao M, Gao J, Wang Y, Yang ZN, Meng X. An *EPFL* peptide signaling pathway promotes stamen elongation via enhancing filament cell proliferation to ensure successful self-pollination in *Arabidopsis thaliana*. New Phytol. 2023:238(3):1045–1058. 10.1111/nph.18806 36772858

[kiae408-B43] Hempel FD , Feldman LJ. Bi-directional inflorescence development in *Arabidopsis thaliana*: acropetal initiation of flowers and basipetal initiation of paraclades. Planta. 1994:192(2):276–286. 10.1007/BF01089045

[kiae408-B44] Hervieux N , Dumond M, Sapala A, Routier-Kierzkowska A-L, Kierzkowski D, Roeder AHK, Smith RS, Boudaoud A, Hamant O. A mechanical feedback restricts sepal growth and shape in *Arabidopsis*. Curr Biol. 2016:26(8):1019–1028. 10.1016/j.cub.2016.03.004 27151660

[kiae408-B45] Honma T , Goto K. Complexes of MADS-box proteins are sufficient to convert leaves into floral organs. Nature. 2001:409(6819):525–529. 10.1038/35054083 11206550

[kiae408-B46] Huang H , Gong Y, Liu B, Wu D, Zhang M, Xie D, Song S. The della proteins interact with MYB21 and MYB24 to regulate filament elongation in *Arabidopsis*. BMC Plant Biol. 2020:20(1):64. 10.1186/s12870-020-2274-0 32033528 PMC7006197

[kiae408-B47] Huijser P , Schmid M. The control of developmental phase transitions in plants. Development. 2011:138(19):4117–4129. 10.1242/dev.063511 21896627

[kiae408-B48] Jaeger J , Irons D, Monk N. Regulative feedback in pattern formation: towards a general relativistic theory of positional information. Development. 2008:135(19):3175–3183. 10.1242/dev.018697 18776142

[kiae408-B49] Jiao Y , Du F, Traas J. The mechanical feedback theory of leaf lamina formation. Trends Plant Sci. 2021:26(2):107–110. 10.1016/j.tplants.2020.11.005 33257261

[kiae408-B50] Junttila O , Hänninen H. The minimum temperature for budburst in Betula depends on the state of dormancy. Tree Physiol. 2012:32(3):337–345. 10.1093/treephys/tps010 22391009

[kiae408-B51] Karabourniotis G , Liakopoulos G, Nikolopoulos D, Bresta P. Protective and defensive roles of non-glandular trichomes against multiple stresses: structure–function coordination. J For Res. 2020:31(1):1–12. 10.1007/s11676-019-01034-4

[kiae408-B52] Kasprzewska A , Carter R, Swarup R, Bennett M, Monk N, Hobbs JK, Fleming A. Auxin influx importers modulate serration along the leaf margin. Plant J. 2015:83(4):705–718. 10.1111/tpj.12921 26111009 PMC4949643

[kiae408-B53] Kierzkowski D , Lenhard M, Smith RS, Kuhlemeier C. Interaction between meristem tissue layers controls phyllotaxis. Dev Cell. 2013:26(6):616–628. 10.1016/j.devcel.2013.08.017 24091013

[kiae408-B54] Kierzkowski D , Runions A, Vuolo F, Strauss S, Lymbouridou R, Routier-Kierzkowska A-L, Wilson-Sánchez D, Jenke H, Galinha C, Mosca G, et al A growth-based framework for leaf shape development and diversity. Cell. 2019:177(6):1405–1418.e17. 10.1016/j.cell.2019.05.011 31130379 PMC6548024

[kiae408-B55] Krizek BA , Meyerowitz EM. The *Arabidopsis* homeotic genes *APETALA3* and *PISTILLATA* are sufficient to provide the B class organ identity function. Development. 1996:122(1):11–22. 10.1242/dev.122.1.11 8565821

[kiae408-B56] Kuchen EE , Fox S, de Reuille PB, Kennaway R, Bensmihen S, Avondo J, Calder GM, Southam P, Robinson S, Bangham A, et al Generation of leaf shape through early patterns of growth and tissue polarity. Science. 2012:335(6072):1092–1096. 10.1126/science.1214678 22383846

[kiae408-B57] Lampugnani ER , Kilinc A, Smyth DR. Auxin controls petal initiation in *Arabidopsis*. Development. 2013:140(1):185–194. 10.1242/dev.084582 23175631

[kiae408-B58] Le Gloanec C , Collet L, Silveira SR, Wang B, Routier-Kierzkowska AL, Kierzkowski D. Cell type-specific dynamics underlie cellular growth variability in plants. Development. 2022:149(14):dev200783. 10.1242/dev.200783 35894230

[kiae408-B59] Li X-M , Jenke H, Strauss S, Bazakos C, Mosca G, Lymbouridou R, Kierzkowski D, Neumann U, Naik P, Huijser P, et al Cell-cycle-linked growth reprogramming encodes developmental time into leaf morphogenesis. Curr Biol. 2024:34:541–556. 10.1016/j.cub.2023.12.050 38244542

[kiae408-B60] Liao CY , Smet W, Brunoud G, Yoshida S, Vernoux T, Weijers D. Reporters for sensitive and quantitative measurement of auxin response. Nat Methods. 2015:12(3):207–210. 10.1038/nmeth.3279 25643149 PMC4344836

[kiae408-B61] Liu T , Longhurst AD, Talavera-Rauh F, Hokin SA, Barton MK. The *Arabidopsis* transcription factor ABIG1 relays ABA signaled growth inhibition and drought induced senescence. Elife. 2016:5:e13768. 10.7554/eLife.13768 27697148 PMC5050019

[kiae408-B62] Mansfield C , Newman JL, Olsson TSG, Hartley M, Chan J, Coen E. Ectopic BASL reveals tissue cell polarity throughout leaf development in *Arabidopsis thaliana*. Curr Biol. 2018:28(16):2638–2646. 10.1016/j.cub.2018.06.019 30100337 PMC6109230

[kiae408-B63] Martignago D , Siemiatkowska B, Lombardi A, Conti L. Abscisic acid and flowering regulation: many targets, different places. Int J Mol Sci. 2020:21(24):9700. 10.3390/ijms21249700 33353251 PMC7767233

[kiae408-B64] Massonnet C , Vile D, Fabre J, Hannah MA, Caldana C, Lisec J, Beemster GTS, Meyer RC, Messerli G, Gronlund JT, et al Probing the reproducibility of leaf growth and molecular phenotypes: a comparison of three *Arabidopsis* accessions cultivated in ten laboratories. Plant Physiol. 2010:152(4):2142–2157. 10.1104/pp.109.148338 20200072 PMC2850010

[kiae408-B65] Maugarny-Calès A , Laufs P. Getting leaves into shape: a molecular, cellular, environmental and evolutionary view. Development. 2018:145(13):dev161646. 10.1242/dev.161646 29991476

[kiae408-B66] Melnyk CW , Schuster C, Leyser O, Meyerowitz EM. A developmental framework for graft formation and vascular reconnection in *Arabidopsis thaliana*. Curr Biol. 2015:25(10):1306–1318. 10.1016/j.cub.2015.03.032 25891401 PMC4798781

[kiae408-B67] Nagpal P , Ellis CM, Weber H, Ploense SE, Barkawi LS, Guilfoyle TJ, Hagen G, Alonso JM, Cohen JD, Farmer EE, et al Auxin response factors ARF6 and ARF8 promote Jasmonic acid production and flower maturation. Development. 2005:132(18):4107–4118. 10.1242/dev.01955 16107481

[kiae408-B68] Nakayama H , Nakayama N, Seiki S, Kojima M, Sakakibara H, Sinha N, Kimuraa S. Regulation of the KNOX-GA gene module induces heterophyllic alteration in North American lake cress. Plant Cell. 2014:26(12):4733–4748. 10.1105/tpc.114.130229 25516600 PMC4311196

[kiae408-B69] Nikovics K , Blein T, Peaucelle A, Ishida T, Morin H, Aida M, Laufs P. The balance between the MIR164A and CUC2 genes controls leaf margin serration in *Arabidopsis*. Plant Cell. 2006:18(11):2929–2945. 10.1105/tpc.106.045617 17098808 PMC1693934

[kiae408-B70] Ori N , Cohen AR, Etzioni A, Brand A, Yanai O, Shleizer S, Menda N, Amsellem Z, Efroni I, Pekker I, et al Regulation of LANCEOLATE by miR319 is required for compound-leaf development in tomato. Nat Genet. 2007:39(6):787–791. 10.1038/ng2036 17486095

[kiae408-B71] Pabõn-Mora N , Sharma B, Holappa LD, Kramer EM, Litt A. The Aquilegia FRUITFULL-like genes play key roles in leaf morphogenesis and inflorescence development. Plant J. 2013:74(2):197–212. 10.1111/tpj.12113 23294330

[kiae408-B72] Pastore JJ , Limpuangthip A, Yamaguchi N, Wu MF, Sang Y, Han SK, Malaspina L, Chavdaroff N, Yamaguchi A, Wagner D. LATE MERISTEM IDENTITY2 acts together with LEAFY to activate APETALA1. Development. 2011:138(15):3189–3198. 10.1242/dev.063073 21750030 PMC3133911

[kiae408-B73] Pelaz S , Tapia-López R, Alvarez-Buylla ER, Yanofsky MF. Conversion of leaves into petals in *Arabidopsis*. Curr Biol. 2001:11(3):182–184. 10.1016/S0960-9822(01)00024-0 11231153

[kiae408-B74] Reeves PH , Ellis CM, Ploense SE, Wu M-F, Yadav V, Tholl D, Chételat A, Haupt I, Kennerley BJ, Hodgens C, et al A regulatory network for coordinated flower maturation. PLOS Genet. 2012:8(2):e1002506. 10.1371/journal.pgen.1002506 22346763 PMC3276552

[kiae408-B75] Rinne PLH , Welling A, Vahala J, Ripel L, Ruonala R, Kangasjärvi J, van der Schoot C. Chilling of dormant buds hyperinduces FLOWERING LOCUS T and recruits GA-inducible 1,3-β-glucanases to reopen signal conduits and release dormancy in Populus. Plant Cell. 2011:23(1):130–146. 10.1105/tpc.110.081307 21282527 PMC3051240

[kiae408-B76] Rodriguez RE , Debernardi JM, Palatnik JF. Morphogenesis of simple leaves: regulation of leaf size and shape. Wiley Interdiscip Rev Dev Biol. 2014:3(1):41–57. 10.1002/wdev.115 24902833

[kiae408-B77] Roeder AHK , Chickarmane V, Cunha A, Obara B, Manjunath BS, Meyerowitz EM. Variability in the control of cell division underlies sepal epidermal patterning in *Arabidopsis thaliana*. PLoS Biol. 2010:8(5):e1000367. 10.1371/journal.pbio.1000367 20485493 PMC2867943

[kiae408-B78] Runions A , Tsiantis M, Prusinkiewicz P. A common developmental program can produce diverse leaf shapes. New Phytol. 2017:216(2):401–418. 10.1111/nph.14449 28248421 PMC5638099

[kiae408-B79] Sablowski R . Control of patterning, growth, and differentiation by floral organ identity genes. J Exp Bot. 2015:66(4):1065–1073. 10.1093/jxb/eru514 25609826

[kiae408-B80] Salem MA , Li Y, Bajdzienko K, Fisahn J, Watanabe M, Hoefgen R, Schöttler MA, Giavalisco P. RAPTOR controls developmental growth transitions by altering the hormonal and metabolic balance. Plant Physiol. 2018:177(2):565–593. 10.1104/pp.17.01711 29686055 PMC6001337

[kiae408-B81] Sapala A , Runions A, Routier-Kierzkowska A-L, Das Gupta M, Hong L, Hofhuis H, Verger S, Mosca G, Li C-B, Hay A, et al Why plants make puzzle cells, and how their shape emerges. Elife. 2018:7:e32794. 10.7554/eLife.32794 29482719 PMC5841943

[kiae408-B82] Sauret-Güeto S , Schiessl K, Bangham A, Sablowski R, Coen E. JAGGED controls *Arabidopsis* petal growth and shape by interacting with a divergent polarity field. PLoS Biol. 2013:11(4):e1001550. 10.1371/journal.pbio.1001550 23653565 PMC3641185

[kiae408-B83] Schiessl K , Kausika S, Southam P, Bush M, Sablowski R. JAGGED controls growth anisotropy and coordination between cell size and cell cycle during plant organogenesis. Curr Biol. 2012:22(19):1739–1746. 10.1016/j.cub.2012.07.020 22902754 PMC3471073

[kiae408-B84] Schinidelin J , Arganda-Carreras I, Frise E, Kaynig V, Longair M, Pietzsch T, Preibisch S, Rueden C, Saalfeld S, Schmid B, et al Fiji: an open-source platform for biological-image analysis. Nat Methods. 2012:9(7):676–682. 10.1038/nmeth.2019 22743772 PMC3855844

[kiae408-B85] Schwarz S , Grande AV, Bujdoso N, Saedler H, Huijser P. The microRNA regulated SBP-box genes SPL9 and SPL15 control shoot maturation in *Arabidopsis*. Plant Mol Biol. 2008:67(1-2):183–195. 10.1007/s11103-008-9310-z 18278578 PMC2295252

[kiae408-B86] Shani E , Ben-Gera H, Shleizer-Burko S, Burko Y, Weiss D, Ori N. Cytokinin regulates compound leaf development in tomato. Plant Cell. 2010:22(10):3206–3217. 10.1105/tpc.110.078253 20959562 PMC2990126

[kiae408-B87] Shikata M , Koyama T, Mitsuda N, Ohme-Takagi M. *Arabidopsis* SBP-box genes SPL10, SPL11 and SPL2 control morphological change in association with shoot maturation in the reproductive phase. Plant Cell Physiol. 2009:50(12):2133–2145. 10.1093/pcp/pcp148 19880401

[kiae408-B88] Silveira SR , Le Gloanec C, Gómez-Felipe A, Routier-Kierzkowska A-L, Kierzkowski D. Live-imaging provides an atlas of cellular growth dynamics in the stamen. Plant Physiol. 2022:188(2):769–781. 10.1093/plphys/kiab363 34618064 PMC8825458

[kiae408-B89] Smyth DR , Bowman JL, Meyerowitz EM. Early flower development in *Arabidopsis*. Plant Cell. 1990:2(8):755–767. 10.1105/tpc.2.8.755 2152125 PMC159928

[kiae408-B90] Strauss S , Runions A, Lane B, Eschweiler D, Bajpai N, Trozzi N, Routier-Kierzkowska A-L, Yoshida S, Rodrigues da Silveira S, Vijayan A, et al Using positional information to provide context for biological image analysis with MorphoGraphX 2.0. Elife. 2022:11:e72601. 10.7554/eLife.72601 35510843 PMC9159754

[kiae408-B91] Su H , Abernathy SD, White RH, Finlayson SA. Photosynthetic photon flux density and phytochrome B interact to regulate branching in *Arabidopsis*. Plant Cell Environ. 2011:34(11):1986–1998. 10.1111/j.1365-3040.2011.02393.x 21726239

[kiae408-B92] Tang H-B , Wang J, Wang L, Shang G-D, Xu Z-G, Mai Y-X, Liu Y-T, Zhang T-Q, Wang J-W. Anisotropic cell growth at the leaf base promotes age-related changes in leaf shape in *Arabidopsis thaliana*. Plant Cell. 2023:35(5):1386–1407. 10.1093/plcell/koad031 36748203 PMC10118278

[kiae408-B93] Vlad D , Kierzkowski D, Rast MI, Vuolo F, Dello Ioio R, Galinha C, Gan X, Hajheidari M, Hay A, Smith RS, et al Leaf shape evolution through duplication, regulatory diversification and loss of a homeobox gene. Science. 2014:343(6172):780–783. 10.1126/science.1248384 24531971

[kiae408-B94] Vuolo F , Kierzkowski D, Runions A, Hajheidari M, Mentink RA, Gupta MD, Zhang Z, Vlad D, Wang Y, Pecinka A, et al LMI1 homeodomain protein regulates organ proportions by spatial modulation of endoreduplication. Genes Dev. 2018:32(21–22):1361–1366. 10.1101/gad.318212.118 30366902 PMC6217736

[kiae408-B95] Wang Y , Strauss S, Liu S, Pieper B, Lymbouridou R, Runions A, Tsiantis Correspondence M. The cellular basis for synergy between RCO and KNOX1 homeobox genes in leaf shape diversity. Curr Biol. 2022:32(17):3773–3784.e5. 10.1016/j.cub.2022.08.020 36029772

[kiae408-B96] Werner S , Bartrina I, Novák O, Strnad M, Werner T, Schmülling T. The cytokinin status of the epidermis regulates aspects of vegetative and reproductive development in *Arabidopsis thaliana*. Front Plant Sci. 2021:12:613488. 10.3389/fpls.2021.613488 33732273 PMC7959818

[kiae408-B97] Whitewoods CD , Gonçalves B, Cheng J, Cui M, Kennaway R, Lee K, Bushell C, Yu M, Piao C, Coen E. Evolution of carnivorous traps from planar leaves through simple shifts in gene expression. Science. 2020:367(6473):91–96. 10.1126/science.aay5433 31753850

[kiae408-B98] Willis L , Refahi Y, Wightman R, Landrein B, Teles J, Huang KC, Meyerowitz EM, Jönsson H. Cell size and growth regulation in the *Arabidopsis thaliana* apical stem cell niche. Proc Natl Acad Sci U S A. 2016:113(51):E8238–E8246. 10.1073/pnas.1616768113 27930326 PMC5187701

[kiae408-B99] Wu W , Du K, Kang X, Wei H. The diverse roles of cytokinins in regulating leaf development. Hortic Res. 2021:8(1):118. 10.1038/s41438-021-00558-3 34059666 PMC8167137

[kiae408-B100] Xu M , Hu T, Zhao J, Park MY, Earley KW, Wu G, Yang L, Poethig RS. Developmental functions of miR156-regulated SQUAMOSA PROMOTER BINDING PROTEIN-LIKE (SPL) genes in *Arabidopsis thaliana*. PLOS Genet. 2016:12(8):e1006263. 10.1371/journal.pgen.1006263 27541584 PMC4991793

[kiae408-B101] Yadav AS , Hong L, Klees PM, Kiss A, He X, Barrios IM, Heeney M, Galang AMD, Smith RS, Boudaoud A, et al Growth directions and stiffness across cell layers determine whether tissues stay smooth or buckle. bioRxiv. 2023.07.22.549953. 10.1101/2023.07.22.549953, 24 July 2023, preprint: not peer reviewed. PMC13581170 42679806

[kiae408-B102] Yang M , Jiao Y. Regulation of axillary meristem initiation by transcription factors and plant hormones. Front Plant Sci. 2016:7:183. 10.3389/fpls.2016.00183 26925087 PMC4757702

[kiae408-B103] Yu S , Lian H, Wang JW. Plant developmental transitions: the role of microRNAs and sugars. Curr Opin Plant Biol. 2015:27:1–7. 10.1016/j.pbi.2015.05.009 26042537

[kiae408-B104] Žádníkova P , Petrášek J, Marhavý P, Raz V, Vandenbussche F, Ding Z, Schwarzerová K, Morita MT, Tasaka M, Hejátko J, et al Role of PIN-mediated auxin efflux in apical hook development of *Arabidopsis thaliana*. Development. 2010:137(4):607–617. 10.1242/dev.041277 20110326

[kiae408-B105] Zhang Z , Runions A, Mentink RA, Kierzkowski D, Karady M, Hashemi B, Huijser P, Strauss S, Gan X, Ljung K, et al A WOX/auxin biosynthesis module controls growth to shape leaf form. Curr Biol. 2020:30(24):4857–4868.e6. 10.1016/j.cub.2020.09.037 33035489

[kiae408-B106] Zhao F , Du F, Oliveri H, Zhou L, Ali O, Chen W, Feng S, Wang Q, Lü S, Long M, et al Microtubule-mediated wall anisotropy contributes to leaf blade flattening. Curr Biol. 2020:30(20):3972–3985.e6. 10.1016/j.cub.2020.07.076 32916107 PMC7575199

