# Supplementary material for: Modulation of cell differentiation and growth underlies the shift from bud protection to light capture in cauline leaves
Source: Plant Physiol. 2024 Aug 6;196(2):1214–30. doi: 10.1093/plphys/kiae408 (PMC11444300; doi:10.1093/plphys/kiae408)
Supplement: kiae408_Supplementary_Data [file kiae408_supplementary_data.zip › kiae408_Supplementary_Data.pdf]

SUPPLEMENTAL DATA

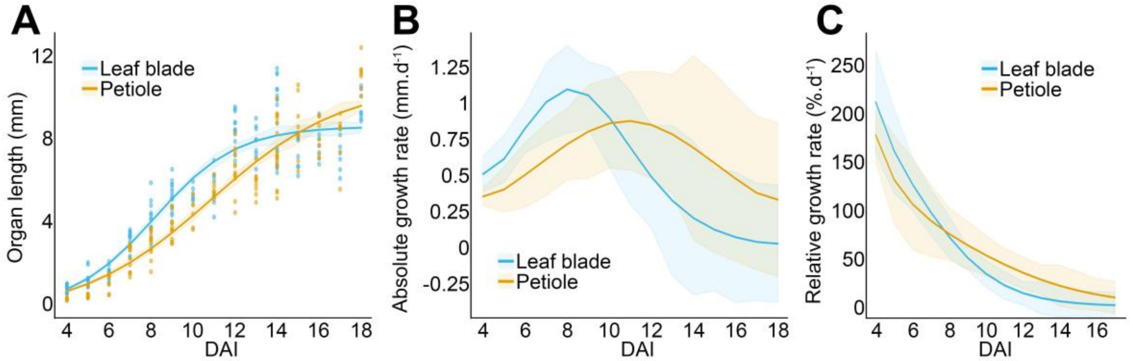

Figure S1. Growth dynamics of the first rosette leaf regions. (A) First rosette leaf blade (blue) and petiole (yellow) length plotted against time from initiation to maturity. Points represent independent samples (n=6-17 individual measurements). (B) Absolute growth rate. (C) Relative growth rate. DAI indicates days after primordium initiation.

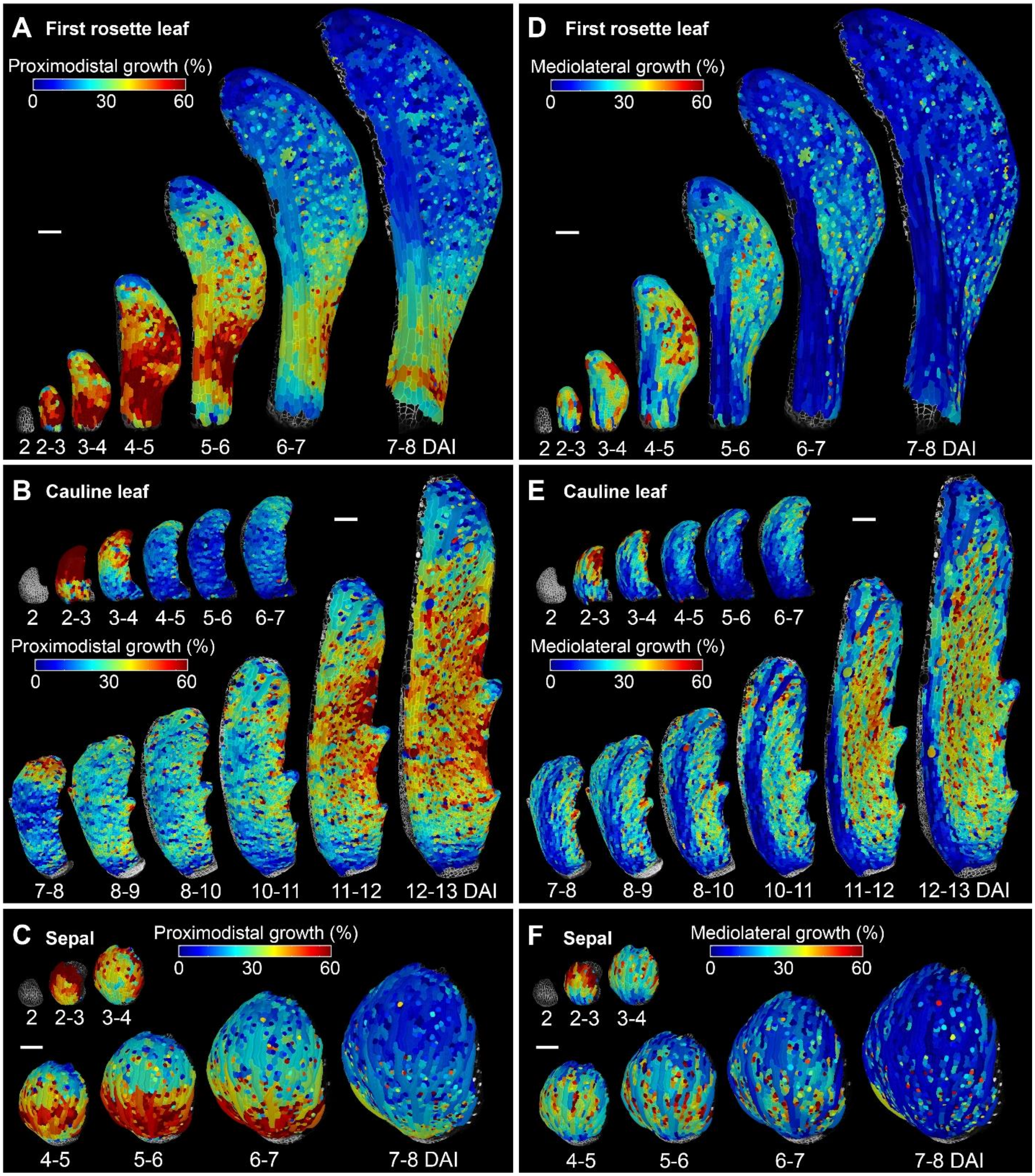

Figure S2. The establishment of the proximodistal growth gradient is delayed in the cauline leaf. (A-C) Heat maps of growth along the proximodistal organ axis for the *Arabidopsis thaliana* first rosette leaf (A), cauline leaf (B), and sepal (C). (D-F) Heat maps of growth along the mediolateral organ axis for the *Arabidopsis thaliana* first rosette leaf (D), cauline leaf (E), and sepal (F). Heat maps generated between two consecutive time points are displayed on the digitally extracted organ surface at the later time point. DAI indicates days after primordium initiation. Scale bars: 100 µm.

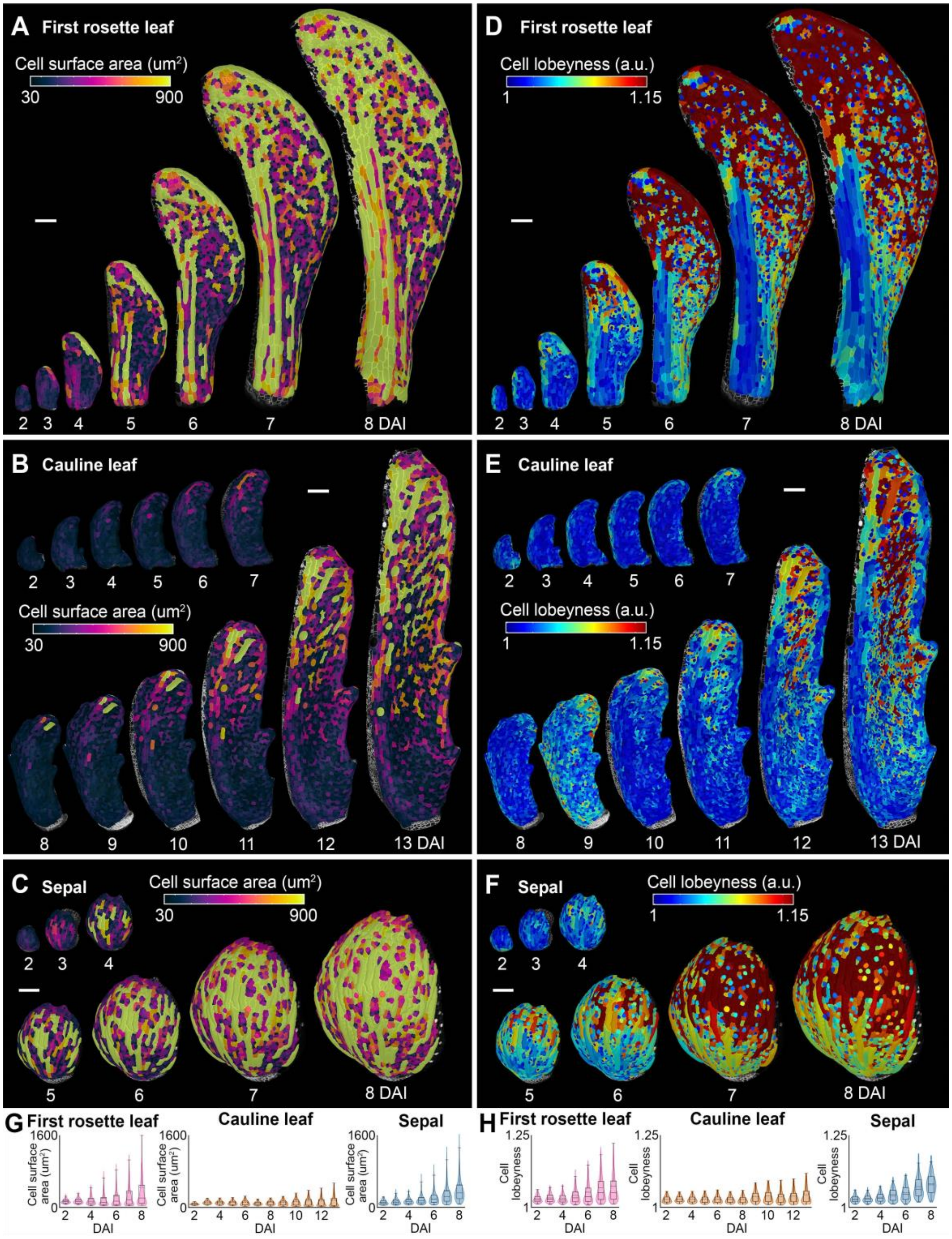

Figure S3. Cauline leaf differentiates at later stages. (A-C) Heat maps of cell surface area for the *Arabidopsis* first rosette leaf (A), cauline leaf (B), and sepal (C). (D-F) Heat maps of cell lobeyness for the first rosette leaf (D), cauline leaf (E), and sepal (F). Heat maps are displayed on the digitally extracted organ surface. Images in panels A to F (organs at 8 DAI) reused in Fig. 4. (G-H) Quantification of cell size (G) and cell lobeyness (H) for the first rosette leaf (left), cauline leaf (middle), and sepal (right). Violin plots and boxplots represent 90% of the values; mean is indicated by a dashed line, median by a line. n=1003-7622 cells (3 independent samples). DAI indicates days after primordium initiation. Scale bars: 100 µm.

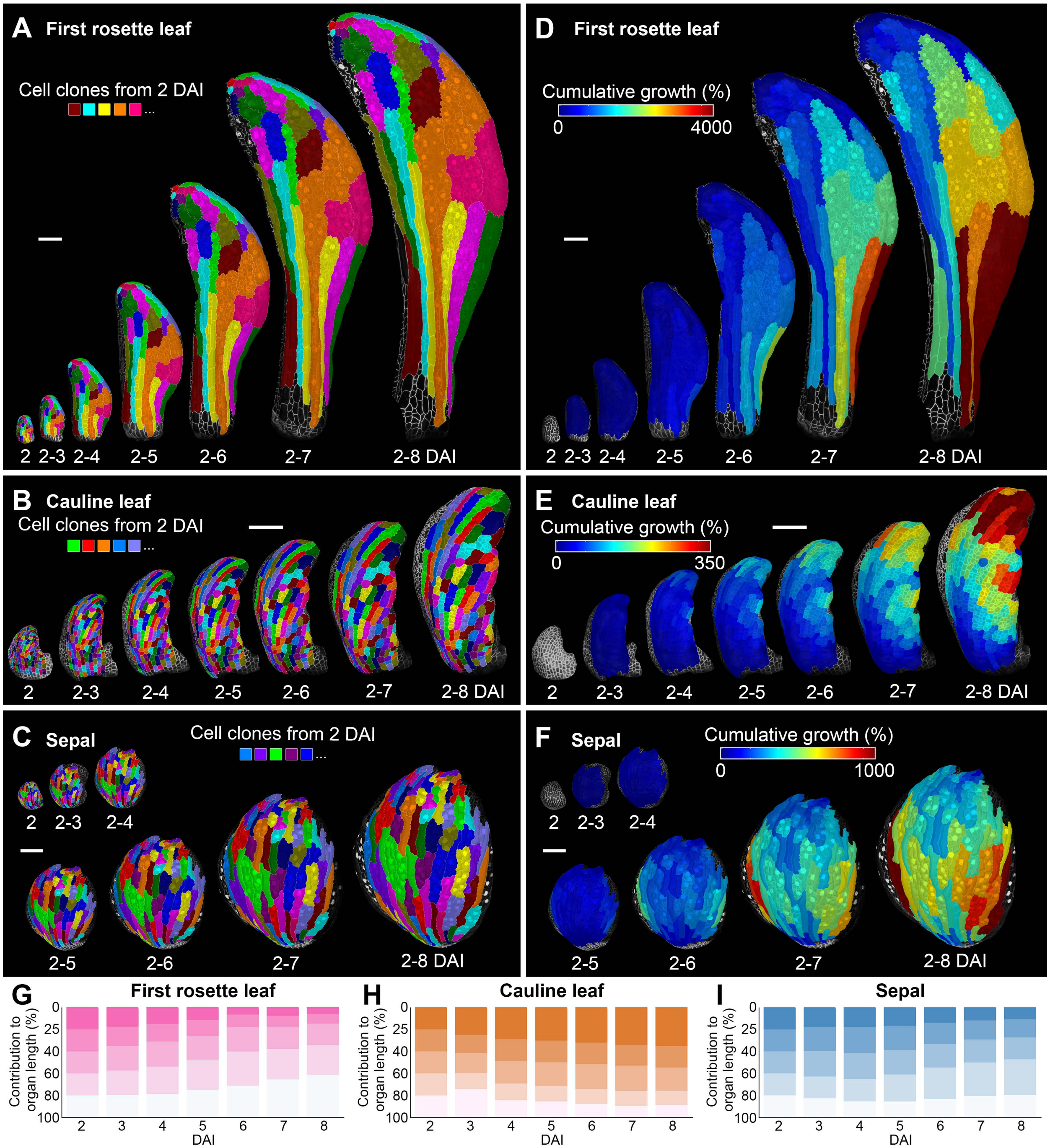

Figure S4. Growth is redistributed distally in the cauline leaf. (A-C) Cell lineage tracing from 2 to 8 days after organ initiation in the first rosette leaf (A), cauline leaf (B), and sepal (C). Colors indicate clones developing from single cells at 2 DAI displayed on the digitally extracted organ surface at the indicated time point. (D-F) Heat maps of cumulative area expansion (from 2 to n DAI) in the first rosette leaf (D), cauline leaf (E), and sepal (F). Heat maps generated between two time points are displayed on the digitally extracted organ surface at the later time point. Images in panels A to F (organs at 6, 10, and 12 DAI) reused in Fig. 5. (G-I) Quantification of the contribution of the clones to the length of the organ between 2 DAI and the indicated time point in the first rosette leaf (G), cauline leaf (H), and sepal (I). Stacked histogram represents the relative contribution of equal segments at 2 DAI to the organ length at n DAI (n=3-7 clones, based on the sample shown in D to F). DAI indicates days after primordium initiation. Scale bars: 100 µm.
