# Supplementary material for: Modulation of cell differentiation and growth underlies the shift from bud protection to light capture in cauline leaves
Source: Plant Physiol. 2024 Aug 6;196(2):1214–30. doi: 10.1093/plphys/kiae408 (PMC11444300; doi:10.1093/plphys/kiae408)
Supplement: kiae408_Supplementary_Data [file pp.00248.2024-s06.pdf › pp.00248.2024-s06.pdf]

Plant Physiology®

Modulation of cell differentiation and growth underlie the shift from bud protection to light capture in cauline leaves

Constance Le Gloanec, Andrea Gómez-Felipe, Viraj Alimchandani, Elvis Branchini, Anne-Lise Routier-Kierzkowska, Daniel Kierzkowski, and Amélie Bauer

Review Timeline:

Submission Date:	13-Feb-2024
Editorial Decision:	18-Mar-2024
Revision Received:	18-Jun-2024
Editorial Decision:	08-Jul-2024
Revision Received:	11-Jul-2024
Accepted:	16-Jul-2024

18-Mar-2024

Dr. Daniel Kierzkowski
Universite de Montreal
Biological Sciences
Montreal, QC
Canada

RE: Modulation of cell differentiation and growth underlie the shift from bud protection to light capture in cauline leaves

Dear Dr. Kierzkowski:

We have received reviews of your manuscript, which you may view at the end of this letter. There is agreement that the work is interesting and novel. On the basis of the advice received and my own evaluation, I believe that, pending revision, this article may ultimately be acceptable for publication in Plant Physiology.

The revised manuscript should address the many helpful suggestions provided by both reviewers. The major concern is centered on the overall descriptive nature of the work. The authors are asked to design some type of experiment that could confirm an importance of the timing of differentiation in cauline leaves or any other key parameter that the authors would choose to focus on. This could include the analysis of a mutant or an external intervention of some sort. Hopefully attempting an experiment is not too onerous and can lead to an improved paper.

When you are ready to submit your revision, click the below link and find the red arrow under your Author Tasks heading for this revision submission.

If you want to visit this version of the manuscript, you may click the link below and then click on the folder that reads "Post Decision Manuscripts".

Link Not Available

When uploading the revised version of this article, please be sure to include as complete an answer as possible to the referees' criticisms during the first round of reviews in the "response to reviewers" section.

Plant Physiology considers a revision for up to 60 days after a manuscript has been provisionally accepted; limited extension of this timeframe is at the discretion of the Monitoring Editor. A reminder notice will be sent to you 2 weeks before the deadline, at which time you may choose to contact the editorial office to request an extension; if so, please provide a justification for your request. After the expiration of this deadline, the article would need to be submitted as a resubmission and would be handled as such.

Thank you very much for giving us an opportunity to review this work. I look forward to receiving the next version.

Sincerely,

Editor, Plant Physiology

Want to add this revision deadline to your calendar? Click below!

-- Reviewer comments:
Reviewer #1 (Comments for the Author):

The manuscript by Le Gloanec et al. analyzes patterns of cell expansion, cell proliferation, and cell differentiation in rosette leaves, cauline leaves, and sepals, and suggests that those patterns lead cauline leaves to behave like sepals at first and remain curled over the inflorescence meristem, presumably to protect it. Later on, those patterns change and cauline leaves

unfold like rosette leaves to maximize their photosynthetic area.

The manuscript is clearly written and well documented. It is, however, mainly descriptive - i.e. no controlled interference was applied to the system to infer mechanisms. Locally interfering with cell expansion, proliferation, and differentiation may be beyond the scope of this manuscript. However, the manuscript ends with the proposal that differences in auxin response or auxin transport between rosette leaves, cauline leaves, and sepals may be underlying the different patterns of cell expansion, proliferation, and differentiation in those three organs. Locally interfering with auxin - production, perception, response, or transport - *is* feasible: for all those pathways, specific inhibitors exist that could be locally applied to developing cauline leaves. For example, the authors' model in Figure 7 seems to predict that applying inhibitors at early stages of cauline leaf development would reduce the curled-up phase and prematurely unfold cauline leaves. It's even possible that premature unfolding of cauline leaves is already observed in single or low-order mutants in auxin pathways that still form cauline leaves (e.g., axr1, tir1;afb2, yuc1;yuc4, pin3;pin4;pin7, etc.) or by growing whole WT plants on concentrations of inhibitors that allow the formation of cauline leaves. And measuring the effects of those types of interference on unfolding of cauline leaves - as opposed to measuring their cell expansion, proliferation, and differentiation - may be sufficient to reveal whether the differences in auxin response and auxin transport the authors observed in rosette leaves, cauline leaves, and sepals are causal to the growth behavior of cauline leaves.

Additional comments (in decreasing order of priority)

1. In the Results section, the authors clearly state that their data only pertain to the abaxial epidermis. Yet throughout the manuscript the authors draw conclusions about the whole leaf, which assumes all the other tissues in the leaf behave as the abaxial epidermis. That assumption is, however, unsupported by available evidence. For example, cell proliferation patterns are different in different tissues of the leaf, and different vascular tissues in the leaf differentiate according to different patterns. I ask for more circumspect wording so as to avoid data over-interpretation or for additional data showing that the other tissues in the leaf behave as the abaxial epidermis.

2. I would like to see more information on how cell proliferation was measured. The authors cite Barbier de Reuille et al., 2015 and Sapala et al., 2018, but I couldn't easily find that information in those two papers. What exactly did the authors measure? What exactly, among the parameters they measured, did they considered a reliable proxy for cell proliferation? Why didn't the authors use known markers of cell proliferation such as CYCB1;1?

3. The authors show in Fig. 1B that cauline leaves can be quite different from one another in both size and shape. I may have missed it, but which cauline leaf was used for all the experiments?

4. The Materials and Methods section mentions that the authors used pUBQ10::myr-YFP and pUBQ10::PM-TDT, but I couldn't find anywhere which line was used to quantify cell expansion, which one was used to quantify cell proliferation, and which one was used to quantify cell differentiation. Please add the missing information.

Minor points

- The pPIN1::PIN1-GFP line described Heisler et al., 2005 is in the Ler background. Did the authors introgress it into Col-0?
- l. 458: "sensing" should read "response" - the authors imaged DR5 not R2D2.

Reviewer #2 (Comments for the Author):

Importance of the findings and scientific merit:
The Authors present a comprehensive quantitative analysis of growth and development of three types of Arabidopsis leaves (sensu lato): rosette leaf, cauline leaf, and sepal, focusing on the cauline leaf as being involved in both photosynthesis (main function of rosette leaves) and protection of reproductive organs (main function of sepal). Based on detailed comparisons of the three organs the Authors draw significant conlusions on relationships between the leaf-like organ growth and funcion as well as the role of auxin in regulation of leaf-like organ morphogenesis. These are novel results. Because the methodology and conclusions are related to leaf functions, development and its regulation, they are significant and will likely be of interest to a wide readership, including developmental biologists, plant physiologists and molecular biologists among others.
Quality of experiments:
The results are based on in vivo imaging mainly in confocal microscopy, which are followed by advanced quantitative analyses using MorphoGraphX and original python codes.
Clarity of presentation:
The presentation is clear and I have just a few suggestions on some aspects that could be considered by the Authors (listed below).

Minor comments in order of their appearance in the text:
1. Line 41-43. Please consider replacing „flat organs" by „laminar organs" (or flattened organs). Sepals even fully developed are definitely not flat but what is common to all the organs that you mention here is their laminar (plate, shell-like) shape

2. Lines 81-96. All this text refers to plants of arabidopsis-like habitus (rosette of leaves, inflorescence with monopodial branching and cauline leaves, etc.) but not to many other plant species. In my opinion it needs to be specified at the beginning of this paragraph.

3. Line 121-122. Rosette leaves also play a protective role for the shoot apex in vegetative developmental phase (as stated later in the text). Why is it not mentioned here?

4. Line 139. „S shape" may be not clear, better reffer again to sigmoid.

5. Figure 1. In my opinion, panels G-I need more labels or explanations in the legend, namely, specifying the leaf base (or tip) and adaxial/abaxial sides.

6. Figure 2,3 etc. Expansion in area or normalised No. of cell divisions are given per day, right? If yes, why this is not on the colorbar or axes legend? It should then be e.g. [% d-1].

7. Line 238. (fig.2 legend). Please rephrase „black dot connected by a full line".

8. Line 285-6. „at 8 DAI" is repeated.

9. Line 287. Do you really mean blade AND margin, or rather blade margin?

10. Line 347 (fig. 5 legend). Should be (D-F).

11. Paragraph starting from line 427. There are also leaves, Populus if I remember, that do not show the proximo-distal growth gradient. Maybe these cases could also be addressed here?

12. Line 499. Do you mean decrease or increase in growth rates?

13. Supplementary figures:
Fig. S2. „area expansion along ... axis" sounds a bit odd. Why not use growth like on figure panels?
Fig. S3. Heat maps show cell surface area, „size" is not a precise term.

Response to reviewer comments for manuscript no. PP2024-RA-00248:

“Modulation of cell differentiation and growth underlie the shift from bud protection to light capture in cauline leaves.”

We would like to thank the reviewers for their thoughtful and constructive comments that helped us improve this manuscript. Below we provide detailed answers to all requests and suggestions.

Reviewer #1 (Comments for the Author):

The manuscript by Le Gloanec et al. analyzes patterns of cell expansion, cell proliferation, and cell differentiation in rosette leaves, cauline leaves, and sepals, and suggests that those patterns lead cauline leaves to behave like sepals at first and remain curled over the inflorescence meristem, presumably to protect it. Later on, those patterns change and cauline leaves unfold like rosette leaves to maximize their photosynthetic area.

The manuscript is clearly written and well documented. It is, however, mainly descriptive - i.e. no controlled interference was applied to the system to infer mechanisms. Locally interfering with cell expansion, proliferation, and differentiation may be beyond the scope of this manuscript. However, the manuscript ends with the proposal that differences in auxin response or auxin transport between rosette leaves, cauline leaves, and sepals may be underlying the different patterns of cell expansion, proliferation, and differentiation in those three organs. Locally interfering with auxin - production, perception, response, or transport - is feasible: for all those pathways, specific inhibitors exist that could be locally applied to developing cauline leaves. For example, the authors' model in Figure 7 seems to predict that applying inhibitors at early stages of cauline leaf development would reduce the curled-up phase and prematurely unfold cauline leaves. It's even possible that premature unfolding of cauline leaves is already observed in single or low-order mutants in auxin pathways that still form cauline leaves (e.g., axr1, tir1;afb2, yuc1;yuc4, pin3;pin4;pin7, etc.) or by growing whole WT plants on concentrations of inhibitors that allow the formation of cauline leaves. And measuring the effects of those types of interference on unfolding of cauline leaves - as opposed to measuring their cell expansion, proliferation, and differentiation - may be sufficient to reveal whether the differences in auxin response and auxin transport the authors observed in rosette leaves, cauline leaves, and sepals are causal to the growth behavior of cauline leaves.

Response #1: Thank you very much for your constructive remarks and suggestions. To support the idea that the delay in auxin response in the leaf blade could underlie the specific developmental trajectory observed in cauline leaves, we performed chemical treatments, as suggested.

First, we treated initiating cauline leaves with ectopic auxin. Cauline leaves sprayed for seven consecutive days with IAA or NAA solution progressed faster through early development, reaching a significantly larger size and straighter shape compared to the mock treatment (see new panels in Fig. 6). This result is consistent with previous time-lapse experiments, where auxin treatment accelerated both cellular growth and differentiation in the adult leaf (Kierzkowski et al., 2019).

We suggested that prolonged expression of PIN1 in the blade of the cauline leaf (Fig. 6B) could prevent auxin accumulation in the abaxial epidermis (Fig. 6A), leading to slower growth and differentiation in this organ compared to rosette leaves. To further test this idea, we blocked the activity of PIN transporters using NPA treatment during the early development of cauline leaves. Similar to the auxin treatment, NPA also accelerated early leaf development, resulting in much larger leaves compared to the mock treatment (see new panels in Fig. 6).

Altogether, these new results suggest that the restriction of auxin spread through the organ epidermis may be the underlying cause of the early developmental delays observed in cauline leaves.

Additional comments (in decreasing order of priority)

1. In the Results section, the authors clearly state that their data only pertain to the abaxial epidermis. Yet throughout the manuscript the authors draw conclusions about the whole leaf, which assumes all the other tissues in the leaf behave as the abaxial epidermis. That assumption is, however, unsupported by available evidence. For example, cell proliferation patterns are different in different tissues of the leaf, and different vascular tissues in the leaf differentiate according to different patterns. I ask for more circumspect wording so as to avoid data over-interpretation or for additional data showing that the other tissues in the leaf behave as the abaxial epidermis.

Response #2: Thank you for highlighting this. Indeed, for technical reasons, we observe only the abaxial epidermis, as the adaxial epidermis is inaccessible for imaging in the cauline leaf. Since cells in plants are connected to each other via cell walls, we do not expect to see very different planar growth rates between the epidermis and adjacent mesophyll cells, as previously shown by Fox et al. (2018). By contrast, cell division could have different dynamics, although they also follow a similar trend, i.e., a basipetal gradient (Fox et al., 2018).

We rephrased the manuscript to better highlight that we observe growth in the abaxial epidermis and to avoid misinterpretation of the data. We also added an additional perspective in the discussion section to emphasize that, in the future, it will be important to extend the study to other tissues of the leaf, including the adaxial epidermis.

2. I would like to see more information on how cell proliferation was measured. The authors cite Barbier de Reuille et al., 2015 and Sapala et al., 2018, but I couldn't easily find that information in those two papers. What exactly did the authors measure? What exactly, among the parameters they measured, did they considered a reliable proxy for cell proliferation? Why didn't the authors use known markers of cell proliferation such as CYCB1;1?

Response #3: We directly and accurately measured the cell proliferation in the abaxial epidermis using lineage tracing tool in MorphoGraphX. It calculates the number of daughter cells (at given time point + 1 day) originating from each mother cells (at previous time point). This is now better described in the method section (Confocal image analysis).

As we directly measure the cumulative cell divisions over the 24h time intervals (all cells originating from one mother cell) we deemed it unnecessary to additionally measure it using

genetic markers that are known to be active at a specific phase of the cell cycle likely underestimating the true cell proliferation rates.

3. The authors show in Fig. 1B that cauline leaves can be quite different from one another in both size and shape. I may have missed it, but which cauline leaf was used for all the experiments?

Response #4: For the quantification of length at the organ scale (Figure 1 and 6), up to two oldest and clearly visible cauline leaves per sample were measured for each sample. For all time lapse-based quantifications, the third cauline leaf was used. We have clarified this in the method section.

4. The Materials and Methods section mentions that the authors used pUBQ10::myr-YFP and pUBQ10::PM-TDT, but I couldn't find anywhere which line was used to quantify cell expansion, which one was used to quantify cell proliferation, and which one was used to quantify cell differentiation. Please add the missing information.

Response #5: We apologize for this omission. We used pUBQ10::myr-YFP line for all time lapse experiments to visualize cell outlines that enabled us to quantify cell expansion, cell proliferation, cell differentiation, and cell lineages. The pUBQ10::myr-TDT was crossed with the pDRv5::nls-3xVENUS reporter to simultaneously visualize cell outlines (TDT) and auxin response (VENUS). This information is now added to the methods section.

Minor points

- The pPIN1::PIN1-GFP line described Heisler et al., 2005 is in the Ler background. Did the authors introgress it into Col-0?

Response #6: Thank you for spotting this error. The pPIN1::PIN1-GFP line is described in Kierzkowski et al., (Dev. Cell 2013) and is in Col-0 background. We corrected this citation in the revised version of the manuscript.

- l. 458: "sensing" should read "response" - the authors imaged DR5 not R2D2.

Response #7: It is now corrected, thank you.

Reviewer #2 (Comments for the Author):

Importance of the findings and scientific merit:

The Authors present a comprehensive quantitative analysis of growth and development of three types of Arabidopsis leaves (sensu lato): rosette leaf, cauline leaf, and sepal, focusing on the cauline leaf as being involved in both photosynthesis (main function of rosette leaves) and protection of reproductive organs (main function of sepal). Based on detailed comparisons of the three organs the Authors draw significant conclusions on relationships between the leaf-like organ growth and function as well as the role of auxin in regulation of leaf-like organ

morphogenesis. These are novel results. Because the methodology and conclusions are related to leaf functions, development and its regulation, they are significant and will likely be of interest to a wide readership, including developmental biologists, plant physiologists and molecular biologists among others.

Quality of experiments:

The results are based on in vivo imaging mainly in confocal microscopy, which are followed by advanced quantitative analyses using MorphoGraphX and original python codes.

Clarity of presentation:

The presentation is clear, and I have just a few suggestions on some aspects that could be considered by the Authors (listed below).

Minor comments in order of their appearance in the text:

1. Line 41-43. Please consider replacing „flat organs" by „laminar organs" (or flattened organs). Sepals even fully developed are definitely not flat but what is common to all the organs that you mention here is their laminar (plate, shell-like) shape.

Response #8: We have replaced “flat organs” with “laminar organs”. Thank you very much for pointing to it.

2. Lines 81-96. All this text refers to plants of arabidopsis-like habitus (rosette of leaves, inflorescence with monopodial branching and cauline leaves, etc.) but not to many other plant species. In my opinion it needs to be specified at the beginning of this paragraph.

Response #9: This is now specified.

3. Line 121-122. Rosette leaves also play a protective role for the shoot apex in vegetative developmental phase (as stated later in the text). Why is it not mentioned here?

Response #10: We agree, and we now mention it in this part.

4. Line 139. „S shape" may be not clear, better reffer again to sigmoid.

Response #11: We now refer to sigmoid only.

5. Figure 1. In my opinion, panels G-I need more labels or explanations in the legend, namely, specifying the leaf base (or tip) and adaxial/abaxial sides.

Response #12: We have now added more explanations in the figure legend.

6. Figure 2,3 etc. Expansion in area or normalised No. of cell divisions are given per day, right? If yes, why this is not on the colorbar or axes legend? It should then be e.g. [% d-1].

Response #13: The time between consecutive time points is usually 1 day (Figures 2 to 4) but can be longer (from 4 to 8 days in Fig. 5). This time, expressed in days after initiation DAI, is

given under each heat map (as done for most of papers contain growth analysis including all previous papers from our labs). To increase clarity, we have modified the figure to precisely indicate what period each heat map represents (1-2, 2-3, 3-4 DAI etc.).

7. Line 238. (fig.2 legend). Please rephrase „black dot connected by a full line".

Response #14: Done.

8. Line 285-6. „at 8 DAI" is repeated.

Response #15: Sorry for this mistake. We have now corrected it.

9. Line 287. Do you really mean blade AND margin, or rather blade margin?

Response #16: We confirm that we mean blade and margin, as lobeyness develop in both regions.

10. Line 347 (fig. 5 legend). Should be (D-F).

Response #17: Thank you for spotting this. We now corrected it.

11. Paragraph starting from line 427. There are also leaves, Populus if I remember, that do not show the proximo-distal growth gradient. Maybe these cases could also be addressed here?

Response #18: You are right, thank you. We added a discussion about and reference to Das Gupta et al., 2015 paper.

12. Line 499. Do you mean decrease or increase in growth rates?

Response #19: We mean decrease. Ectopic overexpression of STM in in the *A. thaliana* leaf leads to a slower growth in the leaf (compare Fig 1A with Fig.4S in Kierzkowski et al., 2019).

13. Supplementary figures:

Fig. S2. „area expansion along ... axis" sounds a bit odd. Why not use growth like on figure panels?

Response #20: We corrected this and now use the term “Growth along the mediolateral/proximodistal organ axis”.

Fig. S3. Heat maps show cell surface area, „size" is not a precise term.

Response #21: We changed “cell size” to “cell surface area”.

08-Jul-2024

Dr. Daniel Kierzkowski
Universite de Montreal
Biological Sciences
Montreal, QC
Canada

RE: Modulation of cell differentiation and growth underlie the shift from bud protection to light capture in cauline leaves

Dear Dr. Daniel Kierzkowski:

We are pleased to accept your manuscript for publication in the Plant Physiology. This acceptance is contingent on revision based on the comments of our reviewers. Both reviewers were impressed with the additional experiments and there are only minor corrections requested from reviewer 2. Thank you for this excellent work.

Please highlight all changes and include a detailed annotation to changes to the text, with line numbers, and noting your responses to the comments.

To submit your revised manuscript, click:

Link Not Available

If you cannot return the revised manuscript within 8 weeks of receipt, please let us know. Otherwise, we will assume that you have elected not to revise the manuscript and are withdrawing it.

Thank you for allowing us to review your work. We look forward to hearing from you soon.

Sincerely,

Editor, Plant Physiology

-- Reviewer comments:
Reviewer #1 (Comments for the Author):

I'm satisfied with how the authors addressed my comments and thank the authors for taking my comments into such consideration.

Reviewer #2 (Comments for the Author):

This is a revised version of the manuscript which I have evaluated as the Reviewer #2. My opinion of the initial submission of this manuscript was positive with minor comments only. Thus I now refer mainly to how the manuscript has been improved. The Authors introduced all the changes which I have suggested. Furthermore, they performed additional experiments, suggested by the Reviewer #1, which made their conclusions even more justified. I now have only two more editorial comments on the revised manuscript: (i) the order of cited literature (page 33) needs correction; (ii) the legend of Fig.6 (line 723) has a style mistake.

Response to reviewer comments for manuscript no. PP2024-RA-00248:

“Modulation of cell differentiation and growth underlie the shift from bud protection to light capture in cauline leaves.”

We would like to thank the reviewers for their thoughtful and constructive comments that helped us improve this manuscript. We now addressed all remining minor issues.

16-Jul-2024
Dr. Daniel Kierzkowski
Universite de Montreal
Biological Sciences
Montreal, QC
Canada

MSID: PP2024-RA-00248R2
MS TITLE: Modulation of cell differentiation and growth underlie the shift from bud protection to light capture in cauline leaves

Dear Dr. Daniel Kierzkowski:

I am pleased to inform you that your revised manuscript "Modulation of cell differentiation and growth underlie the shift from bud protection to light capture in cauline leaves" has been accepted for publication in Plant Physiology. Your manuscript will be checked for consistency with journal formatting and image standards by one of our Science Editors, who will contact you shortly. They may have a number of queries and suggestions for you. Please keep in mind that they work to increase the online visibility of your article and its attraction for readers from the widest of backgrounds. You will have the opportunity to review any revisions, and I hope you will work constructively with them to get the most out of your research. You will also have the opportunity to review all changes when you receive the author proofs for your article.

Once validated for publication, your article will be sent to a Science Editor for a final review of your manuscript and source files. After your final files are uploaded, your article will appear in the next available issue and the online Preview version of your article will be posted after your signed Copyright and Checklist forms and final source files are received.

Please be sure to include the following in your manuscript file:
Harvard style citations
List of all author contributions and funding information at the beginning of manuscript after the titles and author list.
List all supplemental materials associated with the article, with titles, at the end of the ms, before the references and acknowledgments section.
Complete Figure Legends for all figures appearing in the article listed before the Literature Cited.

Thank you and congratulations on your Plant Physiology paper!

Sincerely,
Editor, Plant Physiology

=====
FIRST AUTHOR PROFILES
=====

We recognize the hard work that goes into being First Author of a Plant Physiology paper by publishing a "First Author Feature" for each First Author alongside your paper.

If the first author(s) would like to submit information for a feature on Facebook and Twitter, fill out the form by clicking on the link below:

Plant Physiology First Author Feature Form: https://forms.office.com/r/ZBCGZfhm8F
If you cannot access the form through the link above, please contact Rachel at rbelsky@aspb.org.

To upload an image of yourself to accompany your profile, please put your photo in the correct journal folder at the link below. You must label your image: Author First Name Last Name Article Title.

First Author Profile Image Dropbox: https://www.dropbox.com/scl/fo/xz029p7qjr2jml2rp2kmq/h?dl=0&rlkey=6fkxcaqmxx35ap152b2osy1fz

=====

IMPORTANT REMINDER: PEER REVIEW REPORTS
=====

If you opted to publish a peer review report along with your article during the original submission process, it will be prepared by the editorial staff and publicly posted with your manuscript, inside the zip file that contains any other supplemental material. As a reminder, the peer review report is a public record of all comments from editors and reviewers, as well as your prior responses, as you received them in the decision letters for each draft of your manuscript. If you agreed to publish this report and have changed your mind, or are not sure if you selected this option, please contact the editorial office as soon as possible before signing the license agreement from our publisher.

-- Reviewer comments:
